# mlplasmids: a user-friendly tool to predict plasmid- and chromosome-derived sequences for single species

**DOI:** 10.1099/mgen.0.000224

**Published:** 2018-11-01

**Authors:** Sergio Arredondo-Alonso, Malbert R. C. Rogers, Johanna C. Braat, Tess D. Verschuuren, Janetta Top, Jukka Corander, Rob J. L. Willems, Anita C. Schürch

**Affiliations:** ^1^​ Department of Medical Microbiology, University Medical Center Utrecht, Utrecht, The Netherlands; ^2^​ Julius Centre for Health Sciences and Primary Care, University Medical Centre Utrecht, Utrecht, The Netherlands; ^3^​ Faculty of Medicine, Department of Biostatistics, University of Oslo, Oslo, Norway; ^4^​ Department of Mathematics and Statistics, University of Helsinki, Helsinki, Finland; ^5^​ Infection Genomics, Wellcome Trust Sanger Institute, Hinxton, UK

**Keywords:** whole-genome sequencing, plasmid, chromosome, machine learning, antibiotic resistance

## Abstract

Assembly of bacterial short-read whole-genome sequencing data frequently results in hundreds of contigs for which the origin, plasmid or chromosome, is unclear. Complete genomes resolved by long-read sequencing can be used to generate and label short-read contigs. These were used to train several popular machine learning methods to classify the origin of contigs from *Enterococcus faecium*, *Klebsiella pneumoniae* and *Escherichia coli* using pentamer frequencies. We selected support-vector machine (SVM) models as the best classifier for all three bacterial species (F1-score *E. faecium*=0.92, F1-score *K. pneumoniae*=0.90, F1-score *E. coli*=0.76), which outperformed other existing plasmid prediction tools using a benchmarking set of isolates. We demonstrated the scalability of our models by accurately predicting the plasmidome of a large collection of 1644 *E. faecium* isolates and illustrate its applicability by predicting the location of antibiotic-resistance genes in all three species. The SVM classifiers are publicly available as an R package and graphical-user interface called ‘mlplasmids’. We anticipate that this tool may significantly facilitate research on the dissemination of plasmids encoding antibiotic resistance and/or contributing to host adaptation.

## Data Summary

1. Mlplasmids was implemented as a R package (https://gitlab.com/sirarredondo/mlplasmids) under GNU General Public License v3.0. We additionally developed mlplasmids as a graphical-user interface (https://sarredondo.shinyapps.io/mlplasmids).

2. The complete code and files required to train and benchmark support-vector machine classifiers are publicly available at GitLab (https://gitlab.com/sirarredondo/analysis_mlplasmids).

3. Complete genome sequences from the National Center for Biotechnology Information (NCBI) Assembly Entrez database were used to train and test *E. faecium*, *K. pneumoniae* and *E. coli* mlplasmids classifiers, and their corresponding accession numbers and other details are available in Table S1 (available with the online version of this article).

4. Illumina NextSeq500/MiSeq reads of the 1644 *E. faecium* isolates used in this study are available under the following European Nucleotide Archive (ENA) public project: PRJEB28495.

5. Oxford Nanopore Technologies MinION reads used to complete *E. faecium* genomes are available under the following figshare projects: 10.6084/m9.figshare.7046804, 10.6084/m9.figshare.7047686.

6. Accession numbers from the set of isolates used to benchmark mlplasmids against other tools are available in Table S2.

7. Accession numbers from NCBI draft genome assemblies used to predict the location of the resistome of *E. faecium*, *K. pneumoniae* and *E. coli* are available in Table S3.

**Impact Statement**

Plasmids play a major role in disseminating and facilitating the dissemination of antimicrobial resistance. Whole-genome sequencing is currently used as a standard approach to analyse and study bacterial plasmid sequences. However, the identification of plasmid- and chromosome-derived contigs remains challenging due to the presence of repetitive sequences, which results in a fragmented assembly. Here, we introduce a set of machine-learning classifiers (mlplasmids) that employ pentamer frequencies to predict plasmid- and chromosome-derived contigs from single species. In this study, we show the potential of mlplasmids by accurately predicting the plasmidome content of three relevant bacterial species and highlight mlplasmids′ applicability to predict the location of antibiotic-resistance genes. We have made mlplasmids available as a user-friendly tool that will facilitate the identification of plasmid- and chromosome-derived sequences for large bacterial datasets.

## Introduction

Plasmids are autonomous extra-chromosomal elements that can act as major drivers of variation and adaptation in bacterial populations [1, 2]. Plasmids can also facilitate the dissemination of antimicrobial resistance via horizontal transfer of resistance genes, such as plasmid-derived vancomycin resistance in *E. faecium* or extended-spectrum β-lactamase in Enterobacteriaceae isolates [3–6]. This means that understanding plasmid epidemiology is pivotal to fully understand the introduction and transmission of antimicrobial resistance in bacterial populations [7, 8].

Analysing the plasmid content of large collections of isolates by PCR-based techniques is laborious and has low resolution. Illumina sequencing platforms, which provide short reads (ranging from 150 to 300 bp) with low error rates, have been massively used to assemble bacterial draft genomes [9]. However, the frequent presence of insertion-sequences (IS) and transposable elements in bacterial genomes prohibit their full assembly, because these regions cannot be spanned by short-reads [7, 10]. This results in a fragmented assembly typically consisting of hundreds of chromosomal and plasmid contigs that challenge the inference of the origin of these contigs.

Different tools (PlasmidFinder, cBAR, Recycler, PlasmidSPAdes, PlasFlow) have been proposed to automate the reconstruction of plasmids using short-read whole-genome sequencing (WGS) data [11–15]. However, plasmid predictions are usually incomplete and chromosome-derived contigs are frequently present among the predicted plasmids [16]. This may be partially overcome using tools such as PlacnetW, which allows users to define and solve plasmid boundaries, but limits the high-throughput analysis of short-read WGS data [17, 18].

Long-read WGS has emerged as a solution to obtain complete and error-free plasmid sequences [19, 20]. Read lengths generated by these platforms allow the complete spanning of repeat sequences and obtaining a single contig per replicon [21, 22]. Due to the increasing number of complete genomes available in RefSeq/National Center for Biotechnology Information (NCBI) databases, we explored the possibility of training several popular machine learning algorithms using genome signatures from single-species assemblies. These features have been previously used in cBAR and recently in PlasFlow to distinguish plasmid- and chromosome-derived sequences in metagenomic samples.

Here, we present mlplasmids, a new tool to predict plasmid- and chromosome-derived sequences for a selection of Gram-positive and Gram-negative bacterial species (*E. faecium*, *K. pneumoniae* and *E. coli*) with species-specific classifiers, and we show that mlplasmids outperforms other plasmid prediction tools for these three species. We have made the plasmid models available as an R package and a web-server.

## Methods

### Retrieving complete genome sequences from the NCBI database

We downloaded complete genomes for *E. faecium* (chromosomes=24; plasmids=82), *K. pneumoniae* (chromosomes=156; plasmids=561) and *E. coli* (chromosomes=168; plasmids=415) from the Assembly Entrez NCBI database (https://www.ncbi.nlm.nih.gov/assembly/) with the following criteria: (i) a status level of ‘complete genome’ and (ii) one or more plasmid entries in its respective genome assembly. Retrieved genomes and their corresponding accession numbers are available in Table S1.

### Extending the number of complete genome sequences for *Enterococcus faecium*

Of 1644 *E. faecium* Illumina-sequenced (MiSeq/NextSeq) isolates, 62 isolates were selected based on their preliminary plasmid content using PlasmidSPAdes (version 3.8.2) and presence of known plasmid replication genes [1] (Supplementary Methods S1). We used Oxford Nanopore Technologies (ONT) MinION and hybrid assembly using Unicycler (version 0.4.1) in ‘bold’ mode to obtain complete genome sequences [23].

### Estimating strain diversity in our collection of complete genomes

To ensure that our training and test sets contained chromosome- and plasmid-derived contigs from a diverse set of isolates belonging to each species, we estimated the diversity present in our collection of *K. pneumoniae*, *E. coli* and *E. faecium* genomes with Mash (version 1.1) (sketch size=1000; k-mer=21) [24]. Mash distances were calculated using the total genome content of an isolate (chromosome plus associated plasmids). Computed pairwise Mash distances were transformed into a distance matrix and clustered using the hclust function (method=‘ward.D2’) available in R package stats (version 3.3.3). Hierarchical clustering was visualized using the heatmap.2 function available in R package gplots (version 3.0.1) [25].

### Simulating Illumina sequence reads

To calculate the number of paired reads required to simulate sequence read files, we used wgsim (version 0.3.2, https://github.com/lh3/wgsim) with 50× coverage and no error rate. We retrieved the genome size using bioawk (version 20110810, https://github.com/lh3/bioawk) for each selected complete genome of *K. pneumoniae* and *E. coli*.

### Assembling Illumina sequence reads

Simulated sequence reads of *K. pneumoniae* and *E. coli* were trimmed using seqtk (version 1.2-r94, https://github.com/lh3/seqtk) with the command ‘–trimfq’. We used SPAdes (version 3.6.2) to perform *de novo* assembly [26]. Contigs with a length smaller than 500 bp were excluded.

*E. faecium* Illumina NextSeq reads were trimmed using *nesoni clip*, part of the *nesoni* toolkit (version 0.132), with the following settings: ‘–adaptor-clip yes –match 10 –max-errors 1 –clip-ambiguous yes –quality 10 –length 90 –trim-start 0 –trim-end 0 –gzip no –out-separate yes pairs’. Trimmed reads were then assembled into contigs using SPAdes (version 3.5.0) with default settings. Contigs with a mean coverage lower than 10× and/or a length smaller than 500 bp were removed from the assemblies.

### Labelling short-read contigs as chromosome or plasmid derived

To label contigs as either plasmid or chromosome derived, SPAdes contigs were mapped using bwa-mem (version 0.7.15-r1140) against complete chromosomal and plasmid sequences [27]. Contig alignments were parsed using samtools (version 1.4). This approach allowed to label each SPAdes contig either as plasmid or chromosome derived. SPAdes contigs mapping both to complete chromosomal and plasmid sequences or with a length shorter than 1000 bp were discarded.

### Genomic signatures as features to distinguish plasmid and chromosome sequences

To investigate the role of pentamer frequencies as classifier features to differentiate between plasmid and chromosomal sequences, we retrieved the Assembly Entrez NCBI complete genomes available for *E. faecium*, *K. pneumoniae* and *E. coli* (as previously described in Methods in the section ‘Retrieving complete genome sequences from the NCBI database’). We calculated their pentamer frequencies using the R package biostrings (version 2.42.1) [28] and transformed them into a distance matrix (Euclidean distance). We clustered the resulting matrix using the hclust function (method=‘ward.D2’) from R package stats (version 3.3.3). Hierarchical clustering was visualized using the heatmap.plus function available in R package heatmap.plus (version 1.3). Additionally, we used the t-distributed stochastic neighbour embedding (t-SNE) (theta=0.5, iterations=1000, dims=2, is_distance=TRUE) using the implementation provided in the R package Rtsne (version 0.13) [29].

### Selection of isolates for benchmarking

We excluded a set of isolates from the training set consisting of contigs derived from isolates of *K. pneumoniae* (chromosomes=11; plasmids=33), *E. coli* (chromosomes=3; plasmids=7) and *E. faecium* (chromosomes=7; plasmids=31) for which original Illumina sequencing data and complete genomes were available. Twelve of these isolates were also used in a recent benchmarking publication of plasmid prediction tools [16] (Table S2). From the benchmarking set of isolates, *E. coli* strain K-12 substrain MG1655, *K. pneumoniae* KSB1_7 and *E. faecium* E2079, E2364 and E9101 did not contain any plasmids and were considered as negative controls. None of these data was used to train *E. faecium*, *K. pneumoniae* and *E. coli* mlplasmids models.

### Building a machine-learning model

For each bacterial species, we tuned and compared five different supervised algorithms provided in mlr R package (version 2.11): logistic regression, Bayesian classifier, decision trees, random forest (RF) and support-vector machine (SVM) [30–32]. We defined a two-class classification problem using the category ‘plasmid’ as positive-class. To train and test the resulting classifiers, we considered pentamer frequencies (*n*=1024) that were calculated using the oligonucleotideFrequency function available in R package biostrings (version 2.42.1). The mlr package was used to split SPAdes-labelled contigs into training (80 %) and test sets (20 %), preserving the frequencies of each class in both sets (Supplementary Methods S2 and Table S6).

For *E. faecium* training and test sets, we checked the presence of chromosome-labelled contigs corresponding to plasmid sequences and integrated into the chromosome using blastp (version 2.6.0+) (>60 % coverage, >80 % identity, *E*-value=1×10^−5^) against a curated database of known enterococcal plasmid replication sequences [33]. Decision trees, RF and SVMs hyperparameters were optimized using random search in a predefined search space (Table S5). We performed 10-fold cross-validation to assess the quality of hyperparameters combination, using error rate as a performance measure, except for *E. coli* models in which the true-positive rate was considered to overcome a lower plasmid frequency. For each object, posterior probabilities were generated and the class with a highest posterior probability was assigned.

Optimized classifiers were compared for the test set through receiver operating characteristic (ROC) curves. For each classifier, area under the curve (AUC) and precision-recall curves were calculated to compare resulting classifiers based on different true-positive and false-positive thresholds (from 0 to 1). Metrics were assessed using two different units: number of contigs and sequence size in base pairs. The F1-score was reported to obtain a harmonic mean between specificity and sensitivity. Definitions of the statistics reported in this study are reported below.

$$Sensitivity=\frac{Truepositive\left(contigs/bp\right)}{Truepositive\left(contigs/bp\right)+Falsenegative\left(contigs/bp\right)}$$

$$Specificity=\frac{Truenegative(contigs/bp)}{Truenegative(contigs/bp)+Falsepositive(contigs/bp)}$$

$$Precision=\frac{Truepositive\left(contigs/bp\right)}{Truepositive\left(contigs/bp\right)+Falsepositive\left(contigs/bp\right)}$$

$$Accuracy=\frac{Truepositive(contigs/bp)+Truenegative(contigs/bp)}{Total(contigs/bp)}$$

$$F1-score=\frac{2\times Truepositive\left(contigs/bp\right)}{\begin{array}{c} \times Truepositive\left(contigs/bp\right)+Falsepositive\\ \left(contigs/bp\right)+Falsenegative\left(contigs/bp\right) \end{array}}$$

**Fig. 1.**
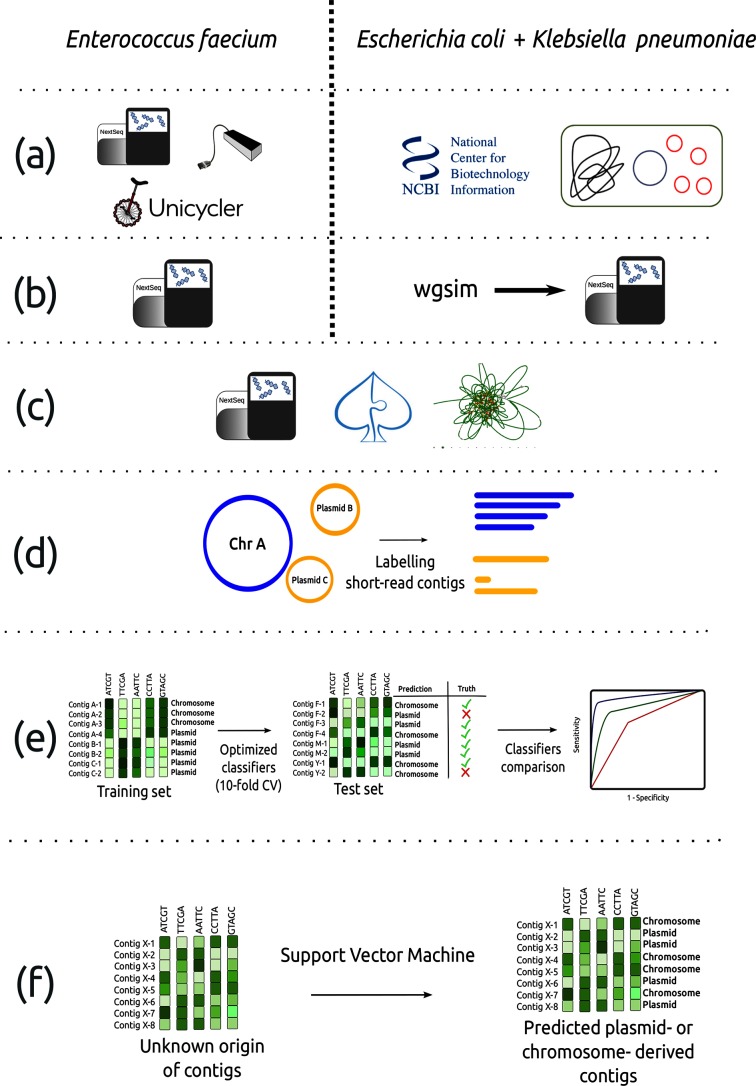
Workflow to create the plasmid models for *Enterococcus faecium, Klebsiella pneumoniae and Escherichia coli*. (a) For *E. faecium*, 62 Illumina-sequenced strains were selected for ONT sequencing and Unicycler was used to extend the number of complete genomes available for this species. For *E. coli and K. pneumoniae*, we downloaded complete genomes with plasmids associated from the Assembly Entrez NCBI database. (b) For *E. coli* and *K. pneumoniae*, we simulated reads with 50× coverage and no error rate using wgsim. (c) Illumina simulated and non-simulated reads were *de novo* assembled using SPAdes. (d) We mapped short-read contigs against complete genome sequences to define a reliable dataset of short-read contigs as plasmid or chromosome derived. (e) For each bacterial species, five machine-learning classifiers were trained (10-fold cross-validation) and compared using a specific bacterial species training and test set. (f) SVM models were implemented in mlplasmids and used to predict plasmid- and chromosome-derived sequences in isolates with only short-read WGS data available. The complete workflow is available from https://gitlab.com/sirarredondo/analysis_mlplasmids.

An overview of the method followed to build the resulting classifiers is shown in Fig. 1.

For each bacterial species, we selected the best model based on the resulting F1-score to predict plasmid- and chromosome-derived sequences. We implemented them in a new R package called mlplasmids available at https://gitlab.com/sirarredondo/mlplasmids under GNU General Public License v3.0. We also developed a Shiny app, available at https://sarredondo.shinyapps.io/mlplasmids/ to enable plasmid prediction with a graphical user interface [34].

### Comparison of mlplasmids against other plasmid prediction tools

We evaluated the performance of mlplasmids against PlasFlow (version 1.0), PlasmidSPAdes (version 3.8.2) and cBar (version 1.2). We considered contigs derived from the isolates described in Methods in the section ‘Selection of isolates for benchmarking’ for which short-read sequencing data and complete genomes were available to validate the presented plasmid prediction tools. cBAR was run using default parameters. PlasFlow was run using standard and recommended parameters corresponding to a minimum posterior probability of 0.7 and minimum contig length of 1000 bp. Contigs with a lower probability were catalogued as ‘unclassified’ by PlasFlow and were excluded from this comparison. PlasmidSPAdes (version 3.8.2) generates its own assembly and the resulting contigs were labelled as true- or false-positive results following the methodology described in Methods in the section ‘Labelling short-read contigs as chromosome or plasmid derived’. For all the tools, we filtered out contigs with a length shorter than 1000 bp.

We benchmarked these plasmid prediction tools using: accuracy, F1-score, and precision. PlasmidSPAdes does not predict chromosome-derived contigs; thus, we could not directly calculate its accuracy and F1-score. To overcome this, we used Quast (version 4.1) to map plasmid-predicted contigs against their respective complete plasmid sequences [35]. We then retrieved the reported ‘genome fraction’ in Quast, which is defined as the percentage of aligned bases from the reference genome covered by contigs predicted as plasmid derived. This allowed us to obtain an estimation of PlasmidSPAdes′ sensitivity [35].

### Validating mlplasmids against complete plasmid sequences

To observe the performance of the resulting classifiers in sequences larger than the mean contig length present in our training and test sets, we used *K. pneumoniae* (*n*=11) and *E. coli* (*n*=3) complete genomes described in Methods in the section ‘Selection of isolates for benchmarking’ to observe mlplasmids performance predicting complete chromosomal and plasmid sequences. In addition, we downloaded complete genomes of *E. faecium* from the Assembly Entrez NCBI database (*n*=24) that were not included in the training set (Table S1).

### Predicting the location of antibiotic-resistance genes

All assemblies of *E. faecium* (*n*=369), *K. pneumoniae* (*n*=1346) and *E. coli* (*n*=5234) with an assembly level corresponding to ‘contig’ were downloaded from NCBI Genomes FTP (ftp.ncbi.nlm.nih.gov/genomes/). For each downloaded draft assembly, we used Abricate (version 0.8.2) (https://github.com/tseemann/abricate) to screen contigs against the ResFinder database (release from 18th May 2016) [36] to determine the presence of antimicrobial-resistance genes. Abricate was run using a minimum DNA identity of 95 % and a minimum coverage of 80 %. To assign a particular contig as plasmid- or chromosome-derived, we used *E. faecium*, *K. pneumoniae* and *E. coli* SVM models in mlplasmids specifying a minimum posterior probability of 0.7 and a minimum contig length of 1000 bp (Table S3).

To validate mlplasmids′ potential to predict the genomic context of a particular antibiotic-resistance gene, we used the isolates described in Methods in the section ‘Selection of isolates for benchmarking’. We used mlplasmids on a contig level to assign whether a particular resistance gene was present on a plasmid or chromosome context. We used identical metrics, introduced in Methods in the section ‘Building a machine-learning model’, to determine performance metrics but considering genes as units.

### Predicting the plasmidome content of *E. faecium*

We used the *E. faecium* optimized model to predict plasmid- and chromosome-derived contigs from the collection of 1644 *E. faecium* Illumina-sequenced (MiSeq/NextSeq) isolates (Table S6). We filtered out contigs with a length shorter than 500 bp and a minimum posterior probability of 0.7 to assign contigs either as plasmid or chromosome derived using the class with a highest posterior probability. SPAdes assembly statistics from this collection are shown in Table S7.

### Data overview

To facilitate the comprehension and reproducibility of the analysis, we summarized in Supplementary Methods S3 the different sequencing and assembly files used in each of the sections previously described in Methods.

## Results

### Diversity of complete genome sequences

To ensure that the new classifiers were built using genome sequences from a large and diverse set of isolates belonging to each species, we first assessed the diversity present in our collections of *E. faecium*, *K. pneumoniae* and *E. coli*. We used Mash to sketch and cluster all retrieved isolates from *E. faecium*, *K. pneumoniae* and *E. coli.* For *E. faecium,* we defined three main clusters and observed that our set of *E. faecium* (*n*=62) extended the diversity present in complete genomes in the Assembly Entrez NCBI database (*n*=24) (Fig. S1). Seven isolates were part of a cluster in which we did not find NCBI complete genomes. Strikingly, we observed a single unique NCBI complete genome forming an independent cluster (GCF_000737555), corresponding to *E. faecium* T110, a probiotic strain (Fig. S1). For *K. pneumoniae*, we also observed and defined three main clusters from all complete genomes available in the Assembly Entrez NCBI database (*n*=156). One of the three clusters was only composed of three *K. pneumoniae* isolates (GCA_000714635, GCF_000019565 and GCF_002156765) and showed a Mash distance higher than 0.05 versus isolates present in the other two major clusters (Fig. S2). For *E. coli*, we observed three major clusters of isolates present in the *E. coli* NCBI collection. All defined *E. coli* clusters presented a high diversity versus each other in terms of Mash distances (Fig. S3).

### Pentamer frequencies differentiate between plasmid- and chromosome-derived sequences in single species

**Fig. 2.**
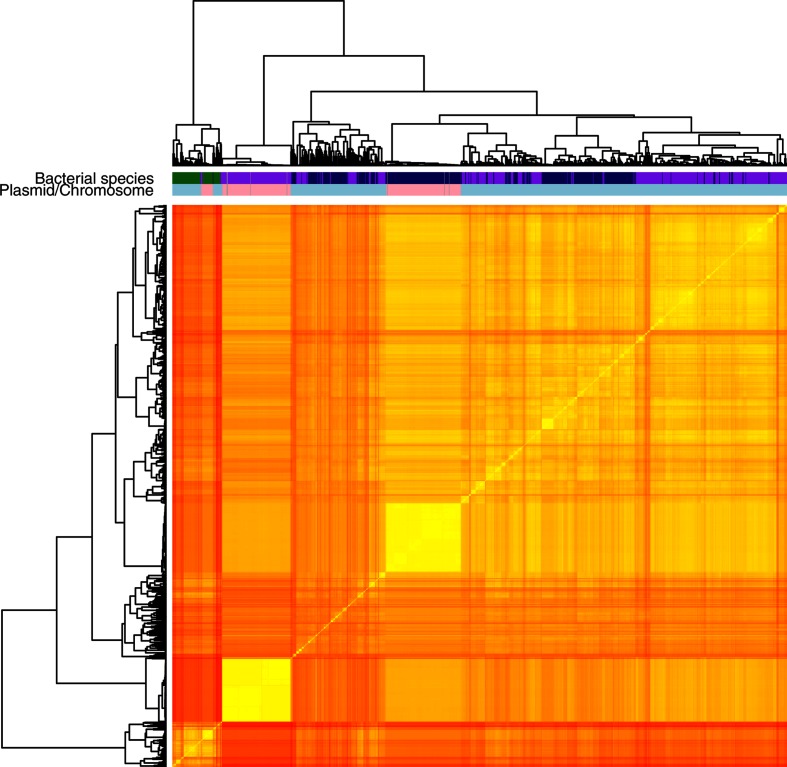
Ward hierarchical clustering of all chromosome and plasmid sequences from the Assembly Entrez NCBI database corresponding to *E. coli, K. pneumoniae* and *E. faecium* based on pentamer frequencies. Each node on the dendrogram corresponds to a either a plasmid (light blue) or chromosome (pink) sequence from *E. coli* (dark blue), *K. pneumoniae* (purple) or *E. faecium* (green).

We investigated the applicability of genomic signatures to distinguish between plasmid- and chromosome-derived sequences by calculating the pentamer frequencies from complete chromosomal and plasmid sequences of *E. faecium, K. pneumoniae* and *E. coli* available in the NCBI database. We then transformed pentamer frequencies into a distance matrix and clustered complete sequences based on their pentamer profile. We observed that pentamer frequencies provided a clear separation between plasmid and chromosome sequences (Figs 2 and S4). However, we observed that chromosome sequences for each species were clustering independently, which suggested that pentamer frequencies differed between bacterial species. Additionally, we observed that plasmid sequences from *E. coli* and *K. pneumoniae* were clustering together, which indicates that plasmids from these two species share a high fraction of k-mers that might be a result of potential plasmid transmission between both species (Fig. 2). We concluded that pentamer frequencies could be used as classifier features to distinguish chromosome and plasmid sequences for single species. In addition, we decided to use exclusively pentamer frequencies for several reasons: (i) the optimal ratio between the number of objects and features (~10) to avoid overfitting problems of the plasmid models due to increase of model complexity, (ii) fast and robust plasmid prediction allowing the possibility of distributing mlplasmids as a graphical-user interface, and (iii) they have been used before to distinguish plasmid sequences in metagenomic samples [12, 15].

### Performance of several popular machine-learning classifiers on single species

**Fig. 3.**
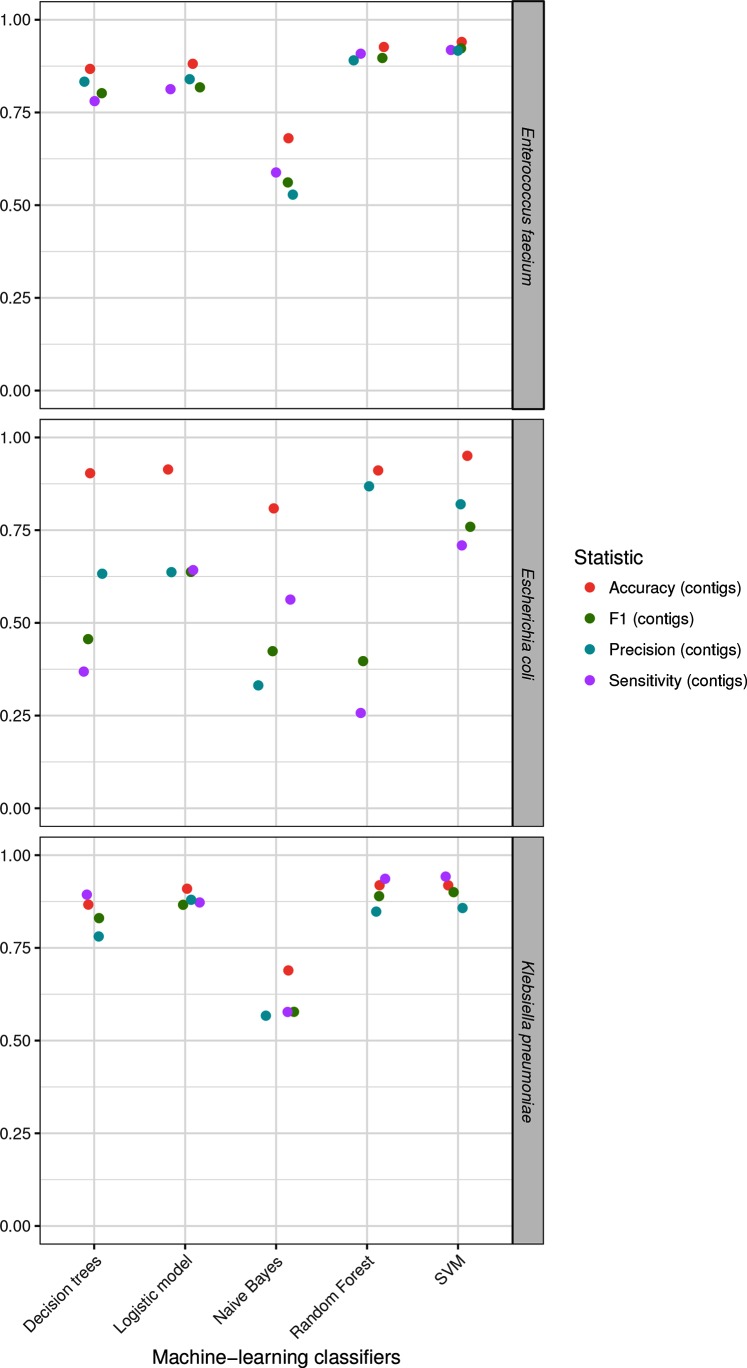
Performance of the optimized machine-learning classifiers. Decision trees, logistic model, Bayesian classifier (naive Bayes), RF and SVM using our test sets for *E. faecium*, *E. coli* and *K. pneumoniae*. The statistics reported are accuracy (red), F1-score (green), precision (blue) and sensitivity (purple), and are indicated using contigs as a performance measure.

SVM was the machine-learning algorithm selected as best classifier for predicting plasmid-derived contigs in the three bacterial species. SVM performance in *E. faecium* (accuracy=0.94; F1-score=0.92) and in *K. pneumoniae* (accuracy=0.92; F1-score=0.90) was better than the other tested machine-learning models and their F1-score, and AUC reflected that prediction of the model was balanced for both classes (Fig. 3). In the case of *E. coli*, SVM performance (accuracy=0.95; F1-score=0.76) reflected that prediction for the plasmid-class was less accurate compared to the chromosome class (sensitivity=0.71) (Fig. 3, Table S8). This can be explained by a lower frequency of the plasmid class (Table S4) present in the training set of the machine-learning classifiers compared to the training sets of *E. faecium* and *K. pneumoniae* or a higher diversity from isolates categorized as belonging to this species (Fig. S4). For the three selected SVM models, we observed that metrics reported were higher when considering base pairs as the unit (Table S8). This indicated that misclassification mostly occurred on short length contigs (<1 kbp) as shown for the *E. faecium* SVM model (Fig. S5).

For the *E. faecium* training and test sets, we checked the presence of chromosome-labelled contigs corresponding to putative integrated plasmids. We observed a low frequency of these contigs (*n*=10 contigs, frequency=0.1). We did not remove them to avoid overfitting problems. After predictions, we observed that the *E. faecium* model predicted two of these contigs as plasmid derived. These two contigs had a small contig length (1.47 and 2.3 kbp). Longer contigs (*n*=8, mean contig length=11.16 kbp) were predicted as chromosome derived. We implemented *E. faecium*, *K. pneumoniae, E. coli* SVM models in a new R package called mlplasmids.

### Benchmarking mlplasmids against existing plasmid prediction tools

**Fig. 4.**
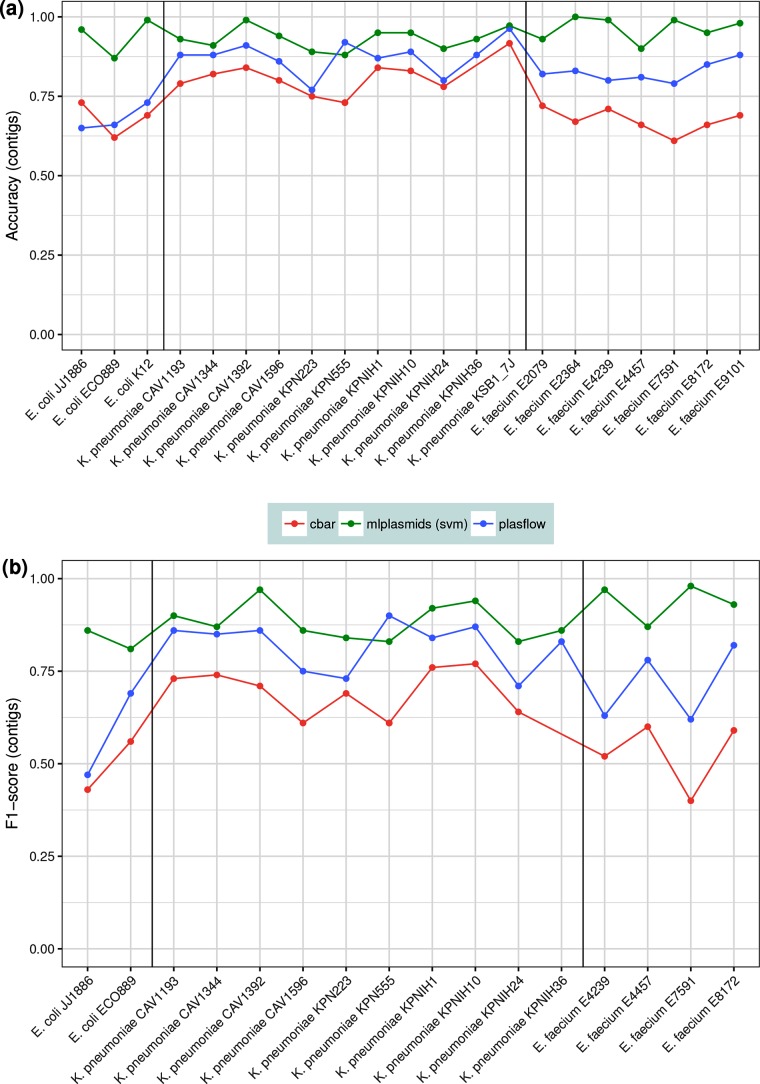
Benchmarking of cBAR (red), mlplasmids (green) and PlasFlow (blue) using an independent set of isolates (*n*=20). (a) Accuracy was measured in contigs and reported for all isolates including samples considered as negative controls (*E. coli* K. 12, *K. pneumoniae* KSB1_7J, *E. faecium* E2079, *E. faecium* E2364 and *E. faecium* E9101). (b) The F1-score was measured in contigs and only reported for isolates bearing plasmids (*n*=16).

**Fig. 5.**
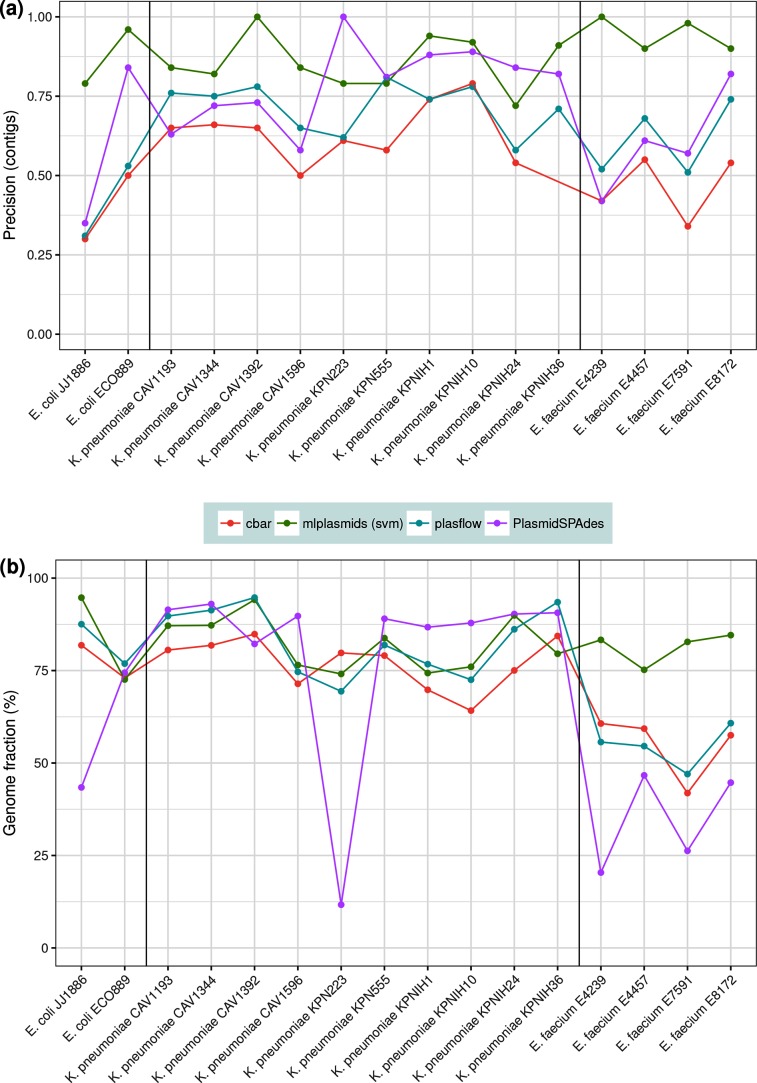
Comparison of cBAR (red), mlplasmids (green), PlasFlow (blue) and PlasmidSPAdes (purple) using an independent set of isolates. (a) Precision was measured in contigs and reported only for isolates bearing plasmids (*n*=16). (b) Genome fraction (measured as percentage of base pairs) was extracted from Quast analysis for isolates bearing plasmids (*n*=16).

We benchmarked mlplasmids against other fully automated plasmid prediction tools using the isolates described in Methods in the section ‘Selection of isolates for benchmarking’ (*E. faecium*=7, *K. pneumoniae*=11, *E. coli*=3). Performance of mlplasmids in *E. faecium* (F1-score=0.94, precision=0.95) was higher than cBAR (F1-score=0.53, precision=0. 46), PlasFlow (F1-score=0.71, precision=0.61) and PlasmidSPAdes (precision=0.61) (Figs 4 and 5). For *E. coli*, mlplasmids performance was superior (F1-score=0.84, precision=0.88) compared to cBAR (F1-score=0.50, precision=0.4), PlasFlow (F1-score=0.58, precision=0.42) and PlasmidSPAdes (precision=0.6). In the case of *K. pneumoniae*, mlplasmids metrics were overall better (F1-score=0.88, precision=0.86) even though performance of PlasFlow (F1-score=0.82, precision=0.72) and PlasmidSPAdes (precision=0.79) was also good, and in the case of *K. pneumoniae* strain KPN555 performance was better compared to mlplasmids (Fig. 5).

The mean genome fraction values of mlplasmids for *E. faecium* (81.5 %), *K. pneumoniae* (82.3) and *E. coli* (83.7 %) indicated that most of the bases from the reference plasmids were covered in the prediction by mlplasmids, even though contigs with a contig length smaller than 1000 bp were filtered out (Fig. 5b). For *K. pneumoniae,* the overall genome fraction of PlasFlow (83.1) was higher than for mlplasmids, but precision (0.72) indicated that a fraction of chromosomal contigs was wrongly predicted as plasmid (Fig. 5a). We further compared mlplasmids and PlasFlow predictions showing the potential of mlplasmids unravelling the origin of contigs unclassified by PlasFlow (Supplementary Results S1, Figs. S6 and S7).

Our approach of training the classifiers on datasets from single species was fundamental to obtain a good precision. This was also reflected in mlplasmids prediction for isolates corresponding to negative controls. For *E. coli* strain K-12 and *K. pneumoniae* KSB1_7J, only a single contig (>1000 bp) was erroneously predicted as plasmid derived. We also observed similar very low numbers of false-positive plasmid assigned contigs for *E. faecium* E2079 (*n*=6) and *E. faecium* E9101 (*n*=1), and for *E. faecium* E2364 (*n*=0) all chromosome-derived contigs were correctly predicted (Fig. 4a).

### Predicting plasmids acquired by horizontal gene transfer

To assess the applicability of mlplasmids detecting plasmids acquired from related species, we considered all the plasmid-derived contigs described in Methods in the section ‘Selection of isolates for benchmarking'. For each dataset of *E. coli*, *K. pneumoniae* and *E. faecium* contigs, we predicted the origin of contigs using all three models available in mlplasmids. As expected, for each dataset the best model to predict chromosome- and plasmid-derived contigs corresponded to the mlplasmids model from the same species (Fig. S8). However, we recovered most of the *E. coli* plasmid contigs (96 %) when using the *K. pneumoniae* model and with an associated high probability of belonging to the plasmid class (mean=0.80) (Fig. S8c). We also observed a similar situation when predicting *K. pneumoniae* plasmid contigs with our *E. coli* model, in which plasmid-derived contigs were detected with a high probability of belonging to that class (mean=0.82) but only 57 % of plasmid-derived contigs were assigned to this category, which could be explained by a lower prevalence of plasmid contigs present during the training of the *E. coli* model (Fig. S8b). This analysis suggested that mlplasmids can correctly predict plasmid sequences transferred to *E. coli* or *K. pneumoniae* coming from a related bacterial species as a result of a horizontal gene transfer event.

However, when using the *E. faecium* model against the *K. pneumoniae* and *E. coli* dataset, we obtained a high number of false-negative contigs and plasmid-derived contigs had a low probability (*K. pneumoniae* mean=0.59; *E. coli* mean=0.62) of belonging to the assigned class (Fig. S8a). This highlighted that pentamer frequencies between chromosome- and plasmid-derived contigs differ between non-related species. Additionally, we refuted the possibility that all sequences predicted with a particular model, but coming from another bacterial species, would have been exclusively assigned to the plasmid class (Fig. S8).

### Applicability for predicting sequences derived from incomplete long-read assemblies

To rule out misclassification of complete plasmid sequences as chromosomal due to a possible correlation of pentamer frequencies and contig length, we evaluated the performance of mlplasmids with chromosomal and plasmid sequences with a sequence length higher than the mean contig size used during the training of mlplasmids classifiers. We predicted complete genome sequences from *E. faecium* (chromosomes=24; plasmids=82), *K. pneumoniae* (chromosomes=11; plasmids=33) and *E. coli* (chromosomes=3; plasmids=7). The observed mlplasmids performance for *E. faecium* (F1-score=0.99), *K. pneumoniae* (F1-score=0.98) and *E. coli* (F1-score=0.92) suggested that mlplasmids can also be used to predict these large contigs correctly. This demonstrates the flexibility of mlplasmids to predict sequences with different lengths compared to the mean contig length used to train the mlplasmids models. Consequently, mlplasmids may facilitate the classification of contigs generated from incomplete hybrid or long-read assemblies as exemplified for isolate *E. faecium* E7070 (Supplementary Results S2, Fig. S9).

### Applicability for predicting the location of antibiotic-resistance genes

To show the potential of mlplasmids in determining whether a particular gene of interest is plasmid or chromosome encoded, we predicted the location of antibiotic-resistance genes in *E. faecium*, *K. pneumoniae* and *E. coli*. Firstly, we determined resistance genes in NCBI draft assemblies by using Abricate to screen contigs against the ResFinder database. Secondly, we used *E. faecium*, *K. pneumoniae* and *E. coli* SVM models in mlplasmids to determine whether these resistance genes were located in plasmid- or chromosome-derived contigs. For each identified resistance gene, we calculated the frequency of finding that particular gene on a predicted plasmid- or chromosome-derived contig.

For *E. faecium*, we assigned a total of 1058 and 1836 genes as chromosome and plasmid located, respectively. We observed that most aminoglycoside-resistance genes [e.g. *ant(6)-Ia_2*] were mainly present in a plasmid context (Fig. S10). Erythromycin-resistance genes were preferentially present in one genomic context depending on the gene variant as exemplified by *erm(A)_1* and *erm(B)*_18 (Fig. S10). As previously described, the *vanA* operons were only present in plasmid-predicted contigs [37]. Furthermore, *vanB* operons were present in both plasmid and chromosomal contexts, but the frequency of chromosome-derived contigs was higher (0.73) (Fig. S10) [38]. Validation of the prediction on *E. faecium* isolates excluded from the model training (*n*=7) revealed that all resistance genes (*n*=43) predicted by Abricate were correctly predicted either as plasmid or chromosome derived (F1- score=1.0).

**Fig. 6.**
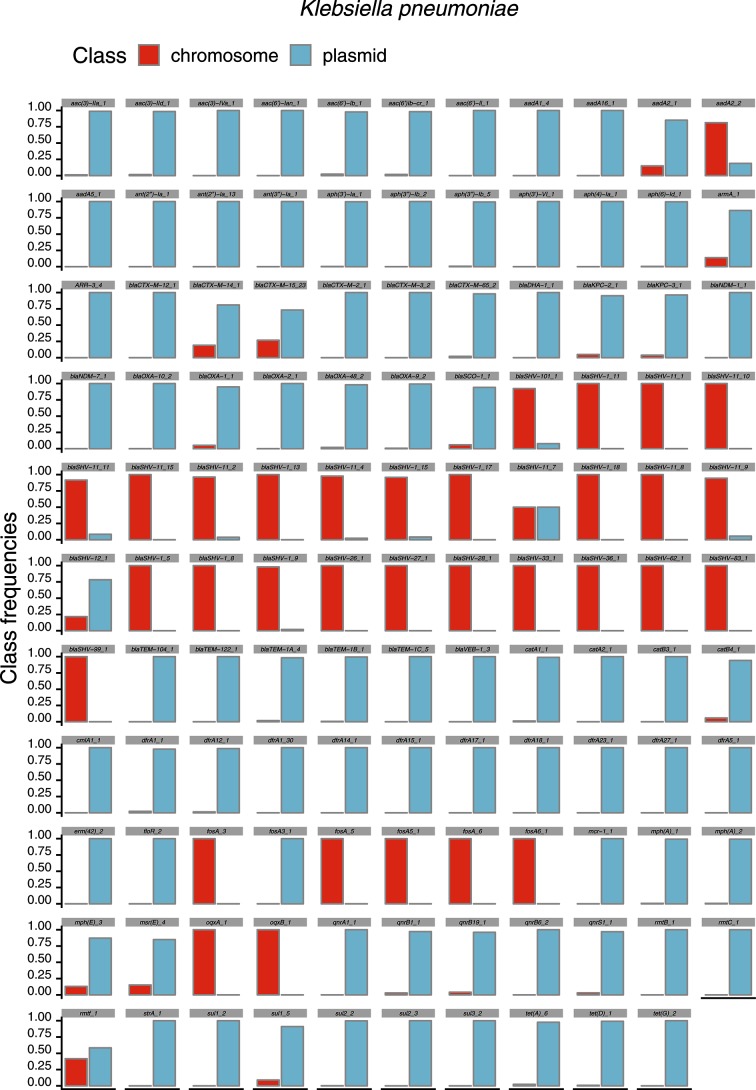
*K. pneumoniae* resistome. Draft genomes available in NCBI Genomes FTP (*n*=1346) were downloaded and screened using Abricate and ResFinder for the presence of antibiotic-resistance genes. Each contig containing a resistance gene was predicted with mlplasmids to have plasmid or chromosome origin. For visualization purposes, only antibiotic-resistance genes present more than five times are shown.

For *K. pneumoniae*, we assigned a total of 5107 and 10 432 ResFinder hits as chromosome and plasmid located, respectively. Most of the antibiotic-resistance genes showed a clear tendency of being present in either a plasmid or chromosomal genomic context (Fig. 6). As described before [39], we observed some notable exceptions, such as *armA* or *bla_CTX-M-14_1_*, in which these particular resistance genes were also present in predicted chromosome-derived contigs (Fig. S11a). We performed the same analysis on *K. pneumoniae* isolates belonging to the independent set (*n*=10) resulting in a total of 41 and 75 genes predicted as plasmid and chromosome encoded, respectively. Mlplasmids evaluation revealed that all predicted plasmid-encoded genes were correctly assigned (precision=1.0) and only five genes were misclassified as chromosome encoded (F1-score=0.96, sensitivity=0.93).

For *E. coli*, we assigned a total 4517 and 8085 ResFinder hits as chromosome and plasmid located, respectively. In contrast to *K. pneumoniae*, we observed that resistance genes were frequently identified in both plasmid and chromosomal contexts (Fig. S12). We also observed differences in gene location between resistance gene variants as exemplified for *qnrS2_1*, which was frequently encoded in predicted plasmid-derived contigs in contrast to *qnrS1_1*, which can be found in both genomic contexts (Fig. S11b). Interestingly, *mcr-1_1* was found in both plasmid and chromosomal contexts in *E. coli,* whereas for *K. pneumoniae* this resistance gene was only identified in plasmid-derived contigs (Fig. S11). Chromosomal locations of *mcr-1_1* for *E. coli* have been described before [40]. We predicted a total of 15 resistance genes from *E. coli* isolates that belonged to the independent set (*n*=3). Mlplasmids performance revealed that four genes that were encoded in a single contig from *E. coli* ECO889 were wrongly predicted as chromosome-encoded, whereas gene assignment was flawless for *Escherichia coli* JJ1886 (F1-score=0.88, sensitivity=0.80). As observed for *Enterococcus faecium* and *K. pneumoniae*, all genes predicted as plasmid encoded were correctly assigned (precision=1.0).

### Applicability for predicting the plasmid content of a single species

Finally, we demonstrate the utility of mlplasmids by predicting the plasmidome content of *E. faecium*. We predicted plasmid-derived sequences from a collection of 1644 Illumina-sequenced *E. faecium* isolates (Table S6). Mlplasmids prediction using our R package took 1 624 509 s (~27 min) on a Linux laptop (Ubuntu 14.04) using a single core. Classifier prediction resulted in 1 94 884 contigs originating from the chromosome and 94 485 contigs with a predicted plasmid origin in 1640 isolates. Mlplasmids did not predict any plasmid-derived contig in four strains, including one of our negative controls (*E. faecium* isolate E2364). The mean posterior probability of the predicted chromosome-derived contigs corresponded to 0.95 versus a mean posterior probability of 0.91 for plasmid-predicted contigs (Fig. S13). This suggested a high likelihood that contigs were correctly assigned to each class. We filtered out contigs with a minimum posterior probability of 0.7 of belonging either to the plasmid or chromosome class to estimate the number of plasmid- and chromosome-derived contigs per isolate. This resulted in mean numbers of ~113 chromosome- and ~52 plasmid-derived contigs per isolate. The mean cumulative length of chromosome- and plasmid- predicted contigs was 2 619 359 and 2 40 324 bp, respectively, which matched with the expected *E. faecium* genome size.

**Fig. 7.**
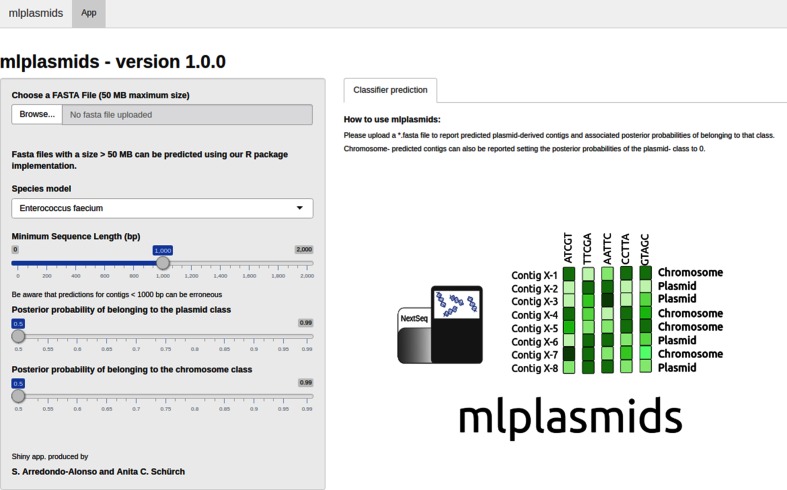
The mlplasmids web-server interface. To facilitate the usability of mlplasmids, we developed a Shiny app, in which users can easily upload single genome assemblies and retrieve mlplasmids prediction.

To facilitate the usability of mlplasmids, we have developed a graphical-user interface in which users can upload and retrieve mlplasmids prediction of genome assemblies online (Fig. 7). As mlplasmids models use pentamer frequencies to predict plasmid-derived contigs, users can collect genome assemblies from several isolates of a single species in a single file, which can facilitate the analysis of a large collection. Assemblies can be uploaded to the web-server as tar.gz files. Users must select the species model (*E. faecium, K. pneumoniae or E. coli*) for the plasmid prediction. After uploading a genome assembly, results appear as tabular data in which each row corresponds to a sequence present in the fasta file. Additionally, results can be filtered using three options: (i) minimum sequence length to report prediction; (ii) minimum posterior probability for assignment of plasmid class; (iii) minimum posterior probability for assignment of chromosome class. Results can be downloaded in csv/xslx format.

## Discussion

We present a set of species-specific machine-learning classifiers to classify plasmid-derived contigs for three clinically relevant species: the Gram-positive bacterium *E. faecium*, and the Gram-negative bacteria *K. pneumoniae* and *E. coli.* We used genomic structure information from complete genomes to label short-read contigs as plasmid- or chromosome-derived, and used them to train and test five different popular machine-learning algorithms.

Genome signatures were previously used in cBAR and more recently for PlasFlow to predict plasmid sequences from primarily metagenomes [12, 15]. In contrast to cBAR and PlasFlow, we trained and benchmarked our SVM classifiers using contigs with a minimum length of 1 kbp. This is important to accurately predict contigs derived from small plasmids (length <5 kbp) or from plasmids with a high number of repeat sequences (e.g. transposons), since this leads to a fragmented assembly with a lower mean contig length. We showed mlplasmids potential to obtain an accurate and reliable plasmidome prediction compared to cBAR and PlasFlow. Furthermore, mlplasmids′ precision when predicting contigs from isolates considered as negative controls was remarkable (Fig. 4a). We have highlighted the potential of mlplasmids to classify the origin of contigs unclassified by PlasFlow. Mlplasmids also outperformed PlasmidSPAdes (Fig. 4), which relies on differences in coverage between plasmids and chromosome in the prediction of plasmid-derived contigs for single genome assemblies. Mlplasmids allows accurate prediction of contigs derived from large plasmids or linear plasmids without differences in sequencing coverage between replicons.

Mlplasmids can predict whether a particular contig is plasmid or chromosome derived. However, it is not possible to cluster plasmid contigs into different bins to observe whether predicted plasmid contigs are derived from the same replicon. Nevertheless, mlplasmids can be used as a basis for plasmid classification by other tools such as PlacnetW [18], facilitating the reconstruction of plasmid sequences in a network graph, or by PlasmidSPAdes filtering of chromosome-derived contigs regardless of contig coverage. Additionally, PlasmidFinder can be used in combination with mlplasmids to find replication genes present in predicted plasmid-derived contigs.

In contrast to cBAR or PlasFlow, mlplasmids is only suitable for genome assemblies from single species. However, we anticipate that a similar methodology can be implemented to create new models for predicting plasmid- and chromosome-derived contigs for other bacterial species with a sufficient number of diverse and complete genomes.

## Data bibliography

1. Arredondo-Alonso S, Schürch AC. Gitlab, https://gitlab.com/sirarredondo/analysis_mlplasmids (2018).
2. Arredondo-Alonso S, Rogers MRC, Braat JC, Verschuuren TD, Top J *et al*. Illumina NextSeq/MiSeq data from 1644 *Enterococcus faecium* isolates, European Nucleotide Archive (ENA) project, PRJEB28495 (2018).
3. Arredondo-Alonso S, Rogers MRC, Braat JC, Verschuuren TD, Top J *et al*. ONT MinION reads used for hybrid assembly with Unicycler, figshare projects: 10.6084/m9.figshare.7046804; 10.6084/m9.figshare.7047686 (2018).
4. Arredondo-Alonso S, Rogers MRC, Braat JC, Verschuuren TD, Top J *et al*. Table S1 (2018).
5. Arredondo-Alonso S, Rogers MRC, Braat JC, Verschuuren TD, Top J, Corander J, Willems RJL, Schürch AC. Table S2 (2018).
6. Arredondo-Alonso S, Rogers MRC, Braat JC, Verschuuren TD, Top J *et al*. Table S3 (2018).

## Supplementary Data

Supplementary File 1

Supplementary File 2
