# Corrigendum: mlplasmids: a user-friendly tool to predict plasmid- and chromosome-derived sequences for single species

**DOI:** 10.1099/mgen.0.000249

**Published:** 2019-01-31

**Authors:** Sergio Arredondo-Alonso, Malbert R. C. Rogers, Johanna C. Braat, Tess D. Verschuuren, Janetta Top, Jukka Corander, Rob J. L. Willems, Anita C. Schürch

**Affiliations:** ^1^​ Department of Medical Microbiology, University Medical Center Utrecht, Utrecht, The Netherlands; ^2^​ Julius Centre for Health Sciences and Primary Care, University Medical Centre Utrecht, Utrecht, The Netherlands; ^3^​ Faculty of Medicine, Department of Biostatistics, University of Oslo, Oslo, Norway; ^4^​ Department of Mathematics and Statistics, University of Helsinki, Helsinki, Finland; ^5^​ Infection Genomics, Wellcome Trust Sanger Institute, Hinxton, UK

There was an error in the Funding information section in the published article.

The Funding information section should include the following sentence: ‘This study was supported by the Joint Programming Initiative in Antimicrobial Resistance (JPIAMR Third call, STARCS, JPIAMR2016-AC16/00039 to SA, RJLW).’

The author apologizes for any inconvenience caused.

