# Supplementary material for: mlplasmids: a user-friendly tool to predict plasmid- and chromosome-derived sequences for single species
Source: Microb Genom. 2018 Nov 1;4(11):e000224. doi: 10.1099/mgen.0.000224 (PMC6321875; doi:10.1099/mgen.0.000224)
Supplement: Supplementary File 1 [file mgen-4-224-s001.pdf]

Supplementary Data

Supplementary Methods S1 (Extending the number of complete genome sequences for *E. faecium*)

Illumina sequencing

Bacterial isolates were grown overnight (O/N) at 37°C on blood agar plates. Single colonies were picked up and grown O/N at 37°C with Brain Heart Infusion (BHI). Bacterial cell pellets were pretreated and incubated 1-4 hours with 180 µL of enzymatic lysis buffer. Subsequently, 0.75 mg proteinase K were added and incubated at 56°C until lysis completion. 20 µL of RNAse A (10mg/mL) were added and incubated for 5’ at room-temperature (RT). Total DNA purification was performed using and following the protocol from NucleoSpin 96 Tissue Core Kit (Machery-Nagel), vacuum processing. DNA concentration was measured using Quant-it Picogreen (Thermo Fisher Scientific). Library preparation was carried out following Nextera DNA Library Prep Reference Guide. Finally, Nextera libraries were sequenced using Illumina NextSeq at USEQ, Utrecht, The Netherlands (http://www.useq.nl).

WGS short-read assemblies

Illumina reads were trimmed using nesoni clip, part of the nesoni toolkit (version 0.132), with the following settings: ‘--adaptor-clip yes --match 10 --max-errors 1 --clip-ambiguous yes --quality 10 --length 90 --trim-start 0 --trim-end 0 --gzip no --out-separate yes pairs:’. Trimmed reads were then assembled into scaffolds using SPAdes (version 3.5.0) with default settings. Scaffolds with an average coverage lower than 10 and/or a length smaller than 500bp were removed from the assemblies.

Isolate selection for ONT sequencing

A fraction (n=60) of the total number of isolates (n=1,644) was selected for long-read sequencing with ONT. The plasmid content of the isolates *in silico* were estimated using PlasmidSPAdes (version 3.8.2) *[1]*. Prokka (version 1.12) was used to annotate the putative plasmid contigs using the *Enterococcus* database included in Prokka [2]. Orthologous clustered genes were estimated using Roary (version 3.8), splitting paralogues and defining a threshold of 95% amino-acid level similarity to cluster protein sequences [3]. This multi-dimensionality matrix was then reduced and visualized to two dimensions using the t-Distributed Stochastic Neighbor Embedding (t-SNE) (theta = 0.5, iterations = 1000, dims = 2) using the implementation provided in the R (version 3.3.3) package Rtsne (version 0.13) [4, 5]. k-means (iter.max = 1000) provided in the R package stats was used to allocate 50 centroids into the dimensionality reduced distribution given by tSNE. Euclidean distance of each isolate was calculated to extract the 50 isolates closest to each centroid.

To cover all plasmid replication genes not present in the first selection, 12 additional isolates were selected for ONT sequencing. This second selection was based on a reciprocal blast of the predicted plasmid orthologous genes against 76 previously described plasmid replication amino-acid sequences from the genus *Enterococcus* [6]. Reciprocal blast allowed to identify miss-annotated genes corresponding to plasmid replication sequences. Isolates bearing plasmid replication genes not present in the first selection were sorted and selected based on highest number of orthologous genes.

ONT sequencing

E. faecium selected isolates were grown O/N at 37°C on blood agar plates, then single colonies were picked up and grown with BHI at 37°C. Genomic DNA was extracted using the Wizard Genomic DNA purification kit (Promega) following manufacturer's instructions. Isolated DNA was sheared (4000 rpm, 2x120 seconds) using G-tubes (Covaris). Library preparation was performed using Ligation Sequencing Kit 1D (SQK-LSK108) with the Native Barcoding Kit 1D (EXP-NBD103). Genomic libraries were loaded onto R9.4 (FLO-MIN106) flowcells using the MinION device (Mk2). Libraries were basecalled using Metrichor workflows (Run 1 ,2, 3), Albacore 1.01 (Run 4, 5) and Albacore 1.1.0 (Run 6). ONT Sequencing and basecalling were conducted at USEQ, Utrecht, The Netherlands (http://www.useq.nl)

ONT reads and hybrid assembly

Fastq files were obtained from base-called data using Poretools (version 0.6.0) except for Run6 in which fastq files were retrieved using Albacore (version 1.1.0). Distribution of read length and total number of reads were calculated using Bioawk (version 20110810, https://github.com/lh3/bioawk). We used Porechop (version 0.2.1, https://github.com/rrwick/Porechop) to trim reads and filter out chimeras from different bins specifying the flag "--discard_middle". Illumina reads were trimmed using seqtk (version 1.2-r94, https://github.com/lh3/seqtk) with the command "--trimfq" prior to assembly.

Hybrid assembly was performed using Unicycler (version 0.4.1), specifying "bold" mode [7]. Briefly, Unicycler uses SPAdes (version 3.6.2) to create different assembly graphs based on different k-mer size only considering Illumina reads [8]. The best assembly graph was selected by Unicycler based on number of dead-ends and contiguity. Next, all ONT reads were used to scaffold and solve the assembly graph. Additionally, we specified the same file as described above (Isolate selection for ONT sequencing) containing 76 known plasmid replication sequences to rotate and change the 0-coordinate of replicons resulting from hybrid assembly [6]. Finally, Unicycler conducted several rounds of Pilon (version 1.22) to polish genome sequences using Illumina reads [9].

Categorization of Unicycler contigs

Unicycler contigs were labeled either as chromosome or plasmids based on size and circularity. Contigs were categorized as chromosome if they were larger than 350 kbp, regardless of circularity.

However, only contigs were categorized as plasmids if they were circular and smaller than 350 kbp. Putative plasmids smaller than 350 kbp and lacking circularization signatures were not categorized. Draft annotation (Prokka - version 1.12) of plasmid sequences allowed to identify and discard four putative complete phage sequences present as circular contigs.

Supplementary Methods S2 (Building a machine-learning model)

For each bacterial species, we tuned and compared five different supervised algorithms provided in mlr R package (version 2.11): logistic regression, Bayesian classifier, decision trees, random forest (RF) and support-vector machine (SVM) [10]. We defined a two-class classification problem using the category 'plasmid' as positive-class. To train and test the resulting classifiers we considered pentamer frequencies (n=1024) which were calculated using oligonucleotideFrequency function available in R package biostrings (version 2.42.1). Mlr package was used to split SPAdes labeled contigs into training (80%) and test set (20%), preserving the frequencies of each class in both sets (Supplementary Table S4).

Decision trees, random forest and support-vector machines hyperparameters were optimized using random search in a predefined search space (Supplementary Table S5). We performed 10-fold cross-validation to assess the quality of hyperparameters combination, using error rate as performance measure. For each object, posterior probabilities were generated and the class with a highest posterior probability was assigned.

Supplementary Methods S3

In this study, we used Illumina NextSeq/MiSeq data for 1,644 *E. faecium* isolates that are available under the ENA project PRJEB28495. A fraction (n = 62) of these 1,644 *E. faecium* isolates was completed using ONT MinION reads which are publicly available under the figshare projects: 10.6084/m9.figshare.7046804 ; 10.6084/m9.figshare.7047686

From these 62 ONT isolates, 5 were not used to label short-read contigs to train and test mlplasmids models. These 5 isolates (E2079, E2364, E4457, E7591, E8172 and E9101) were used to benchmark *E. faecium* mlplasmids models against other plasmid tools. A complete overview of the different datasets used in this study is available at Supplementary Table S6.

Supplementary Results S1

<u>Comparison of mlplasmids against other plasmid prediction tools</u>

Only with the purpose of comparing mlplasmids and plasflow prediction, we created an artificial and third category for mlplasmids named 'unclassified' in which we included all contigs first assigned as

plasmid- or chromosome-derived but with a posterior probability lower than 0.7. We only defined a mlplasmids ‘unclassified’ category for this particular analysis, since mlplasmids prediction only consists of two classes: plasmid or chromosome, and users can decide whether filter out predicted contigs based on their associated posterior probabilities.

For the three single species datasets, the frequency of this category was lower for mlplasmids (*E. faecium* = 0.03 ; *K. pneumoniae* = 0.10 ; *E. coli* = 0.05) compared to PlasFlow (*E. faecium* = 0.16; *K. pneumoniae* = 0.17 ; *E. coli* = 0.21) (Fig. S6). These results showed that most of predicted contigs had an associated high posterior probability of belonging to either plasmid- or chromosome-class with mlplasmids compared to PlasFlow. To show the potential of mlplasmids predicting unclassified contigs from PlasFlow, we considered unclassified contigs by plasflow and observed the posterior probabilities given by mlplasmids. For *E. faecium* and *K. pneumoniae* datasets, we observed that unclassified contigs from PlasFlow derived from the chromosome-class and plasmid-class were mostly correctly predicted by mlplasmids (Fig. S7a and S7b). For *E. coli*, unclassified contigs from the plasmid-class showed a non-uniform distribution whereas contigs from the chromosome-class were in general correctly predicted (Fig. S7c).

Supplementary Results S2

<u>Applicability for predicting sequences derived from incomplete long-read assemblies</u>

For all bacterial species, mlplasmids did not recover any false positive sequences (Specificity = 1). For *E. coli*, only a single plasmid sequence (NC_022662.1) was wrongly predicted as chromosome-derived but with a low posterior probability associated to that class (0.53). In the case of *K. pneumoniae*, mlplasmids misclassified a plasmid sequence with a length of 26.45 kbp (NZ_CP015133.1) from *K. pneumoniae* strain KPN555. For *E. faecium* two sequences were misclassified as chromosomal (NZ_LT598665.1 and NZ_CP019991.1) and the last sequence (NZ_CP019991.1) could correspond to a phage since its NCBI annotation showed two phage-related genes. This demonstrates the flexibility of mlplasmids to predict sequences with different lengths compared to average contig length used to train and test resulting classifiers and discarded misclassifications due to a correlation between pentamer frequencies and contig length. This may facilitate the classification of contigs generated from incomplete hybrid or long-read assemblies as exemplified for isolate *E. faecium* E7070. This isolate was selected for ONT sequencing and after hybrid assembly, 16 contigs were reported. Contigs predicted as plasmid by mlplasmids (n = 6) contained circularization signatures whereas the rest of the contigs (n = 10) were predicted as chromosome-derived (Fig. S9). This facilitated the design of appropriate PCR reactions to complete the genome sequence for E7070.

Supplementary Tables

Supplementary Table S4. Description of the training and test sets used for each bacterial species. For each dataset (training or test), the number of objects (SPAdes contigs) and number of features (5-mer combinations) are indicated.

Bacterial species	Set	Number of objects	Number of features	Prevalence plasmid-class	Prevalence chromosome-class
E. faecium	Training set	8336	1024	0.33	0.67
E. faecium	Test set	2085	1024	0.34	0.66
K. pneumoniae	Training set	10051	1024	0.38	0.62
K. pneumoniae	Test set	2513	1024	0.37	0.67
E. coli	Training set	10061	1024	0.12	0.88
E. coli	Test set	2651	1024	0.14	0.86

Supplementary Table S5. Hyperparameters optimized for decision trees, random forest, and support vector machine.

Classifier	Hyperparameter	Search space (min-max value)
Decision trees	minsplit	10/50
Decision trees	minbucket	5/50
Decision trees	cp	0.001/0.2
Random Forest	ntree	50/1000
Random Forest	mtry	3/10
Random Forest	nodesize	10/50
Support-vector machine	C	(-10)/10
Support-vector machine	sigma	(-10)/10

Supplementary Table S6.Sequencing/Assembly data used in this study.

Bacterial species	Analysis	Dataset	Availability
E. faecium	Labeling short-read contigs as chromosome- or plasmid-derived	Newly generated *E. faecium* genomes (n = 55)	Illumina NextSeq/Miseq reads: ENA Project : PRJEB28495<br><br>ONT MinION reads:<br>figshare projects:<br>10.6084/m9.figshare.704680410<br>.6084/m9.figshare.7047686
E. faecium	Benchmarking against other plasmid tools	Newly generated *E. faecium* genomes (n = 7)	Illumina NextSeq/Miseq reads; ENA Project : PRJEB28495<br><br>ONT MinION reads;<br>figshare projects:<br>10.6084/m9.figshare.7046804<br>10.6084/m9.figshare.7047686
E. faecium	Prediction of the plasmidome content	Newly generated 1,644 *E. faecium* genomes	Illumina NextSeq/Miseq reads; ENA Project : PRJEB28495
E. faecium	Validating mlplasmids against complete genome sequences	Suppl. Table S1	Publicly available NCBI genomes
E. faecium	Predicting the location of AMR genes	Suppl. Table S3	Publicly available NCBI genomes
K. pneumoniae and *E. coli*	Labeling short-read contigs as chromosome- or plasmid-derived	Suppl. Table S1	Publicly available NCBI genomes
K. pneumoniae and *E. coli*	Benchmarking against other plasmid tools	Suppl. Table S2	Publicly available NCBI genomes
K. pneumoniae and *E. coli*	Predicting the location of AMR genes	Suppl. Table S3	Publicly available NCBI genomes

Supplementary Table S7. SPAdes assembly statistics using Illumina MiSeq/NextSeq.

Technology	Number of isolates	Mean Coverage	Mean N50	Mean contig length	Median contig length	Average Number of contigs
Illumina MiSeq	63	98 X	54616 bp	21531 bp	6898 bp	169.1
Illumina NextSeq	1581	113 X	52256 bp	17989 bp	5356 bp	176.3

Supplementary Figures

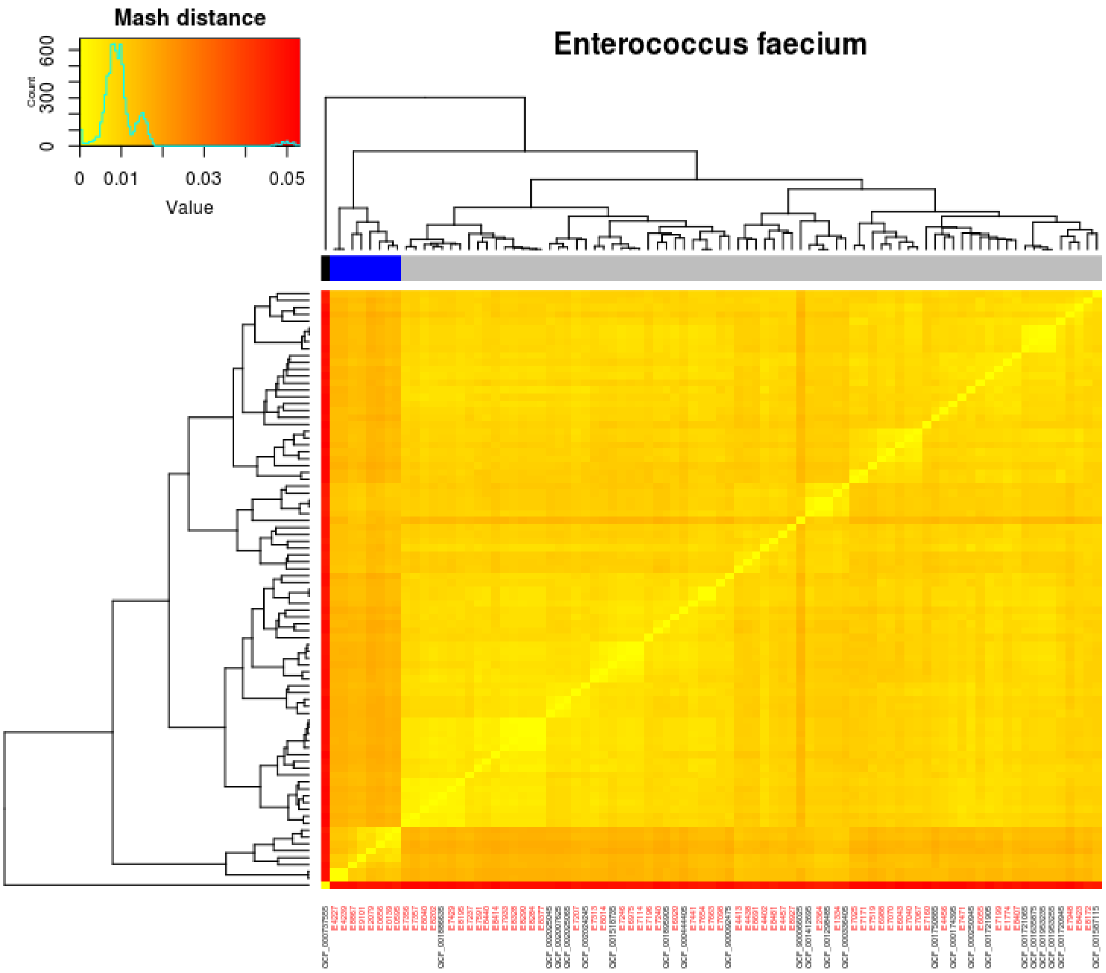

Figure S1. Ward hierarchical clustering of computed pairwise mash distances (k = 21 ; s = 1,000) from *E. faecium* isolates. Based on dendrogram branch lengths, we defined three clusters (black, blue and grey) and visualized mash distances using heatmap based on their genome content similarity. At the bottom y-axis, we coloured in red *E. faecium* isolates (n = 60) that were selected and completed using ONT sequencing and Illumina sequencing. Rest of the isolates corresponded to publicly available NCBI complete genomes from *E. faecium* (n = 24).

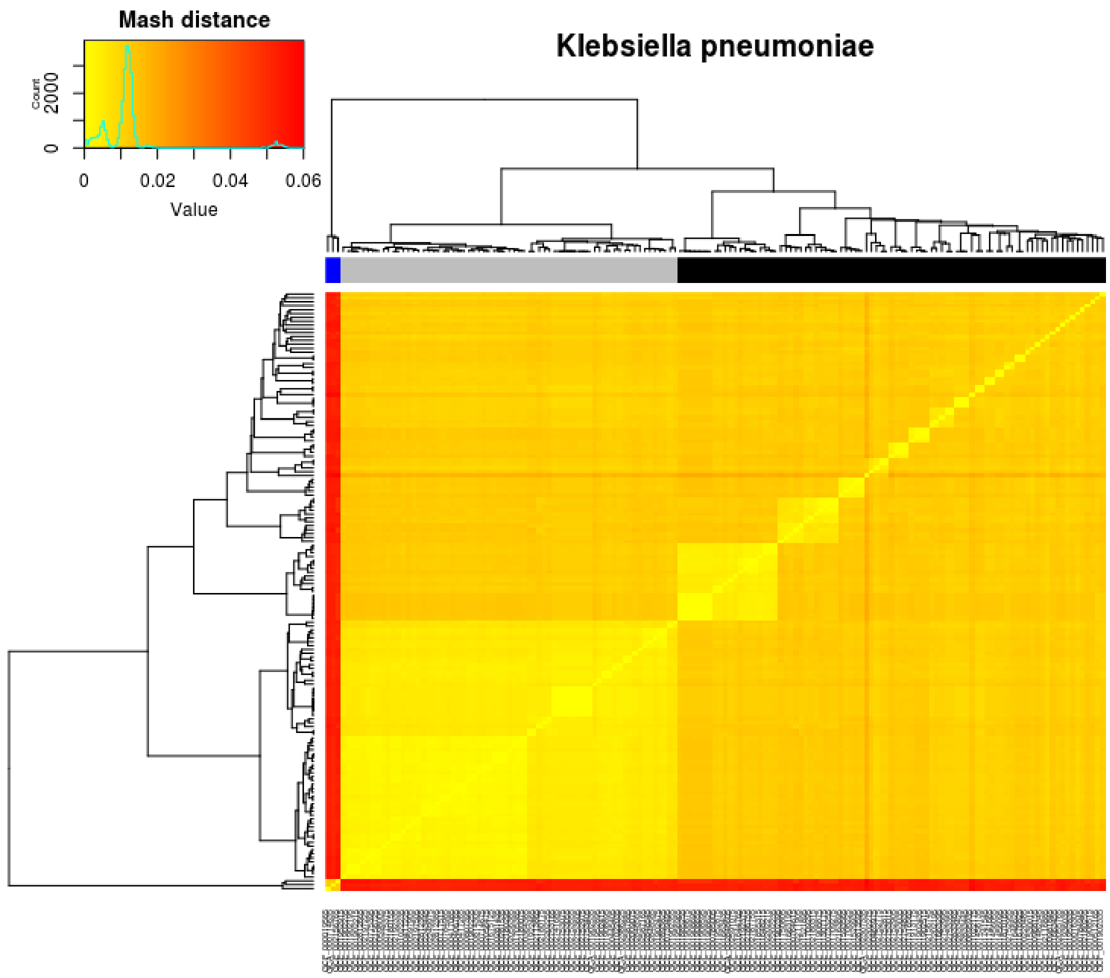

Figure S2. Ward hierarchical clustering of computed pairwise mash distances (k = 21 ; s = 1,000) from *K. pneumoniae* isolates retrieved from Assembly Entrez NCBI database (n = 156). Based on dendrogram branch lengths, we defined three clusters of isolates (blue, grey and black) and visualized mash distances using heatmap to group isolates based on their genome content similarity.

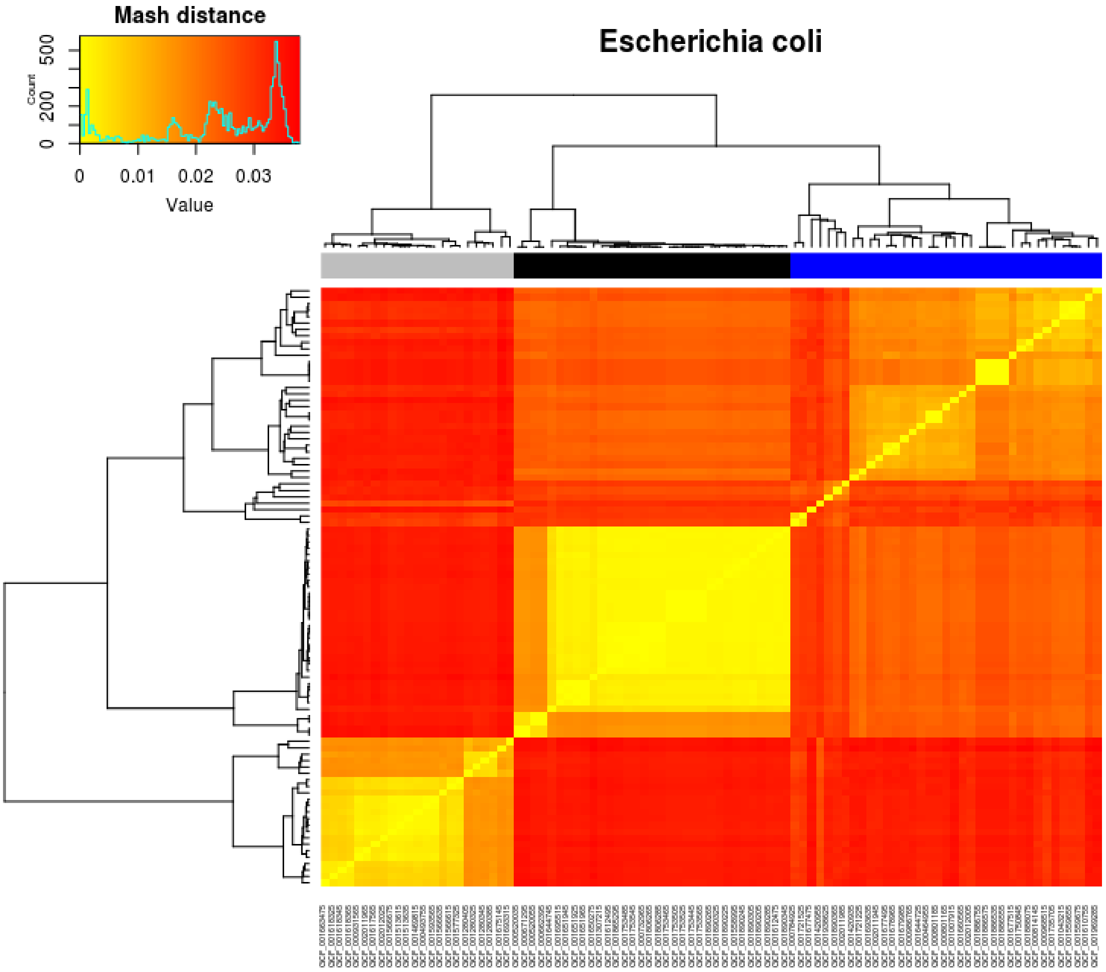

Figure S3. Ward hierarchical clustering of computed pairwise mash distances (k = 21 ; s = 1,000) from *E. coli* isolates retrieved from Assembly Entrez NCBI database (n = 168). Based on dendrogram branch lengths, we defined three clusters of isolates (grey, black and blue) and visualized mash distances using heatmap to group isolates based on their genome content similarity.

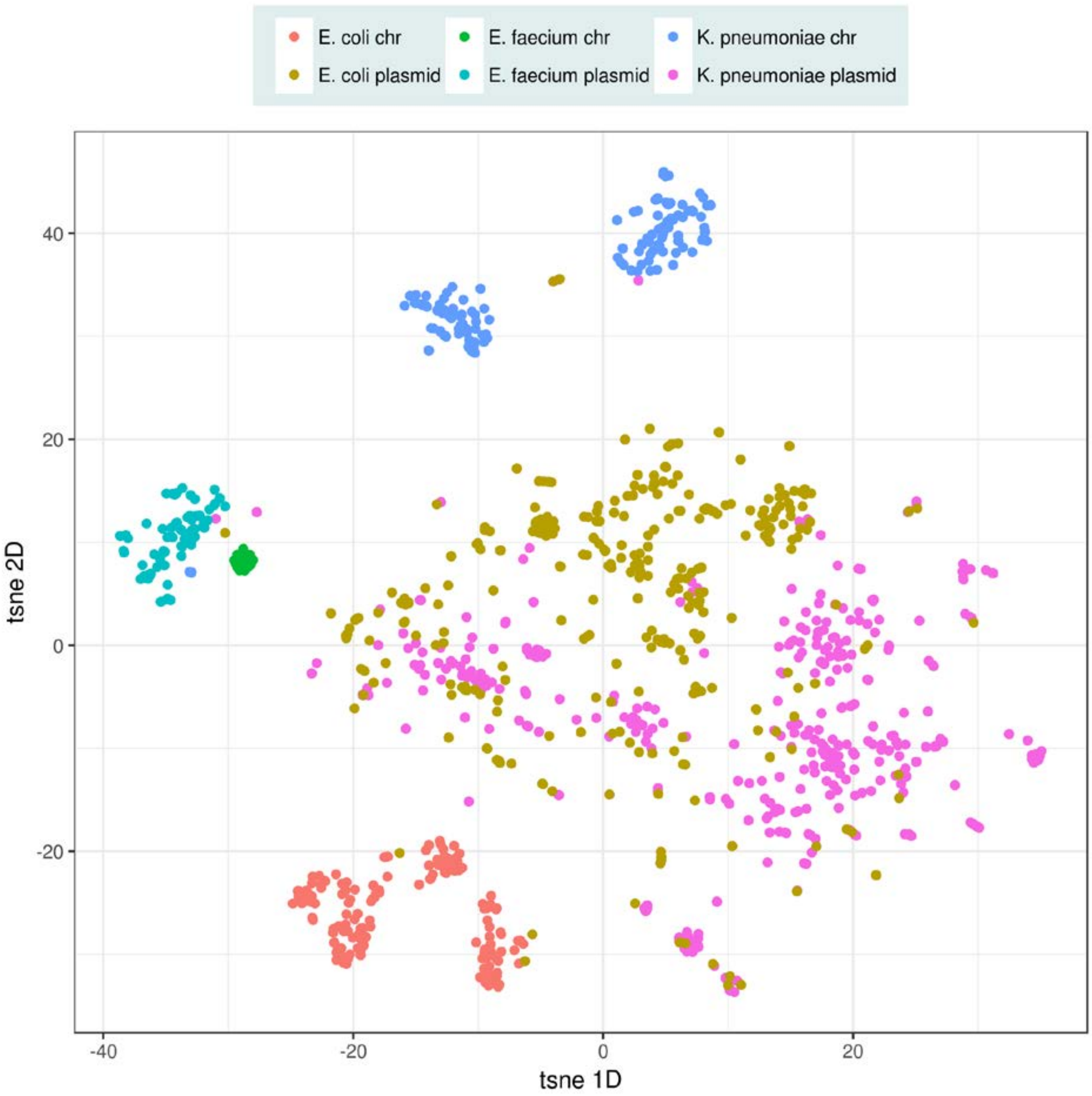

Figure S4. t-sne clustering of all chromosome and plasmid sequences from Assembly Entrez NCBI database corresponding to *E. coli, K. pneumoniae* and *E. faecium* based on pentamer frequencies. Each point in the graph corresponds to a different type replicon: *E. coli* chromosome (red), *E. coli* plasmid (yellow), *K. pneumoniae* chromosome (dark blue), *K. pneumoniae* plasmid (pink), *E. faecium* chromosome (green) and *E. faecium* plasmid (light blue).

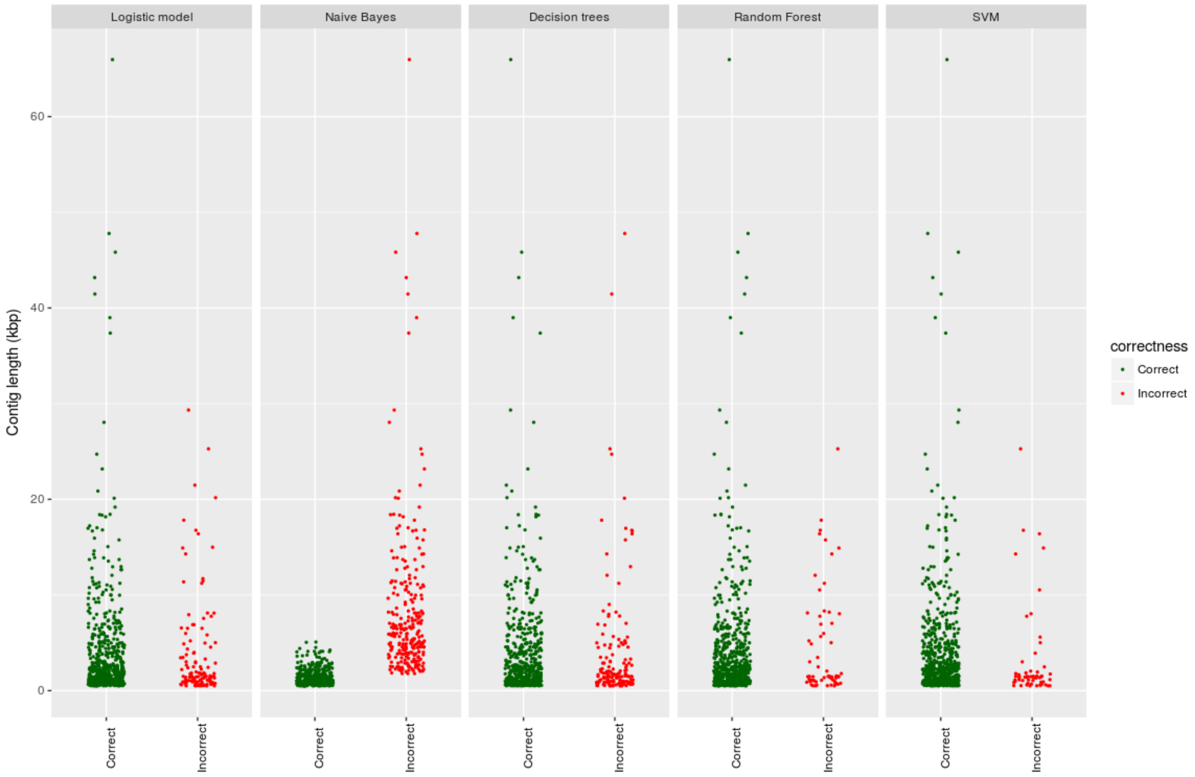

Figure S5. Distribution of correct- and miss- classified short-reads contigs for: Logistic Model, Bayesian Classifier (Naive Bayes), Decision trees, Random Forest, and Support-Vector Machine (SVM). Except for the Bayesian classifier, misclassification most notably occured in contigs with

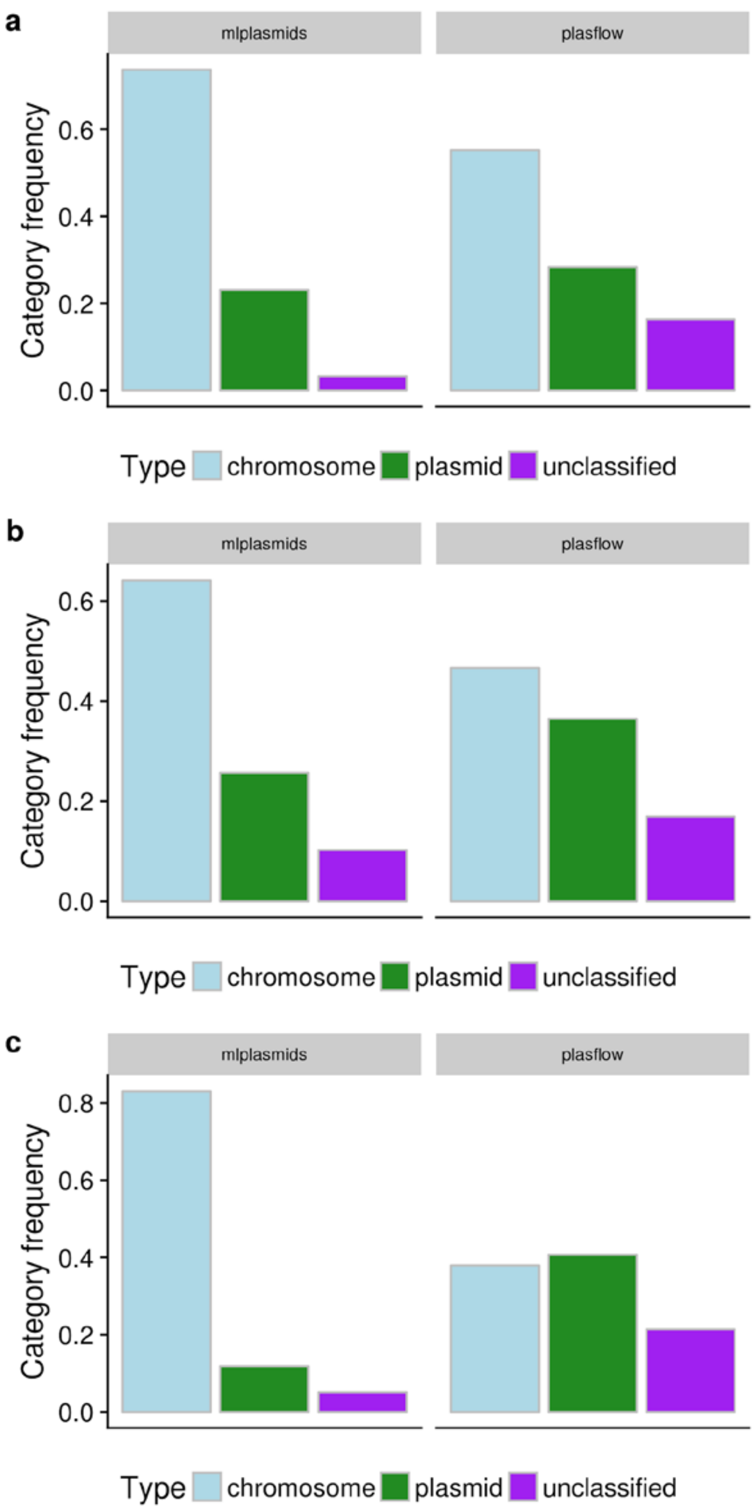

Figure S6. Categorizing the prediction of mlplasmids and plasflow for *E. faecium* (a), *K. pneumoniae* (b) and *E. coli* (c) contigs belonging to our validation sets. We used a minimum posterior probability of 0.7 to assign a contig either to the chromosome- or plasmid-class and with a minimum length of 1,000 bp. Rest of the contigs were included in the category 'unclassified'.

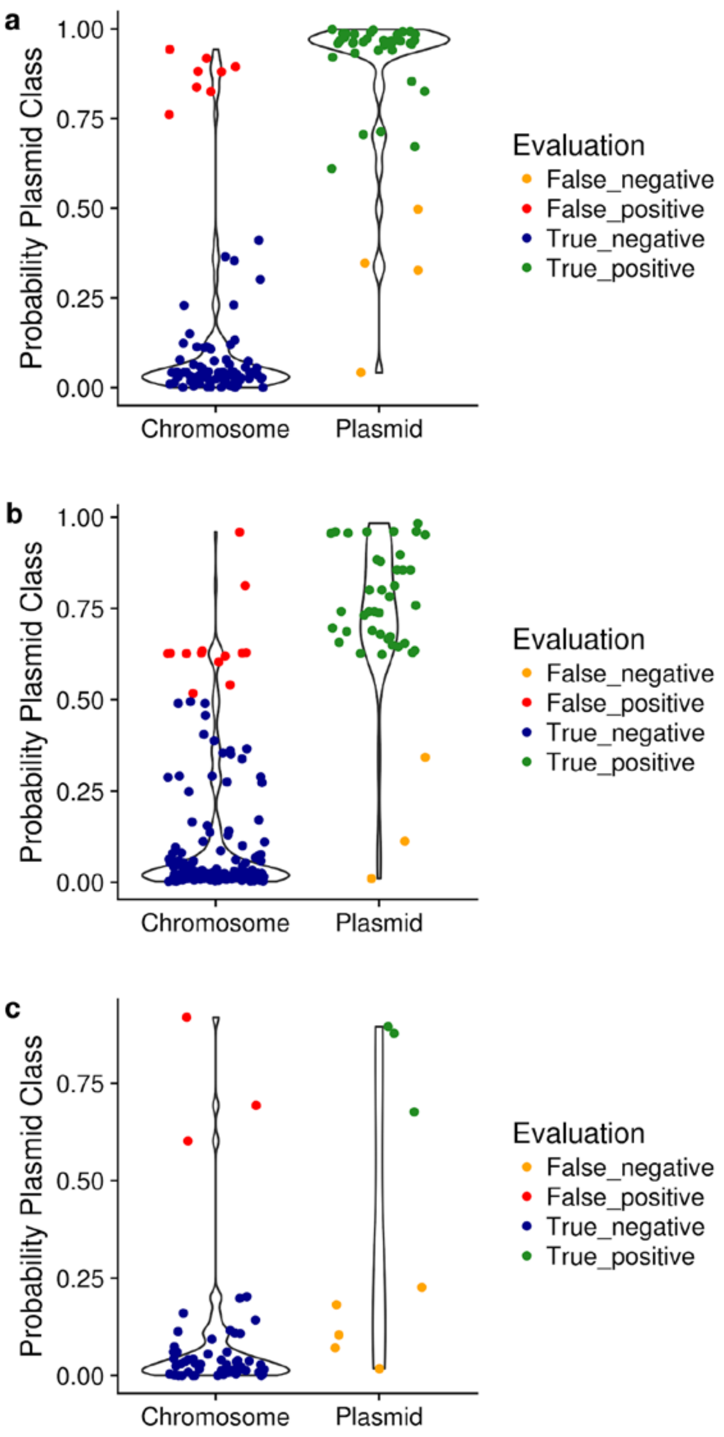

Figure S7. Unraveling the origin of contigs unclassified by plasflow using mlplasmids. *E. faecium* contigs (a), *K. pneumoniae* (b) and *E. coli* (c) which were predicted as 'unclassified' by plasflow were interrogated using mlplasmids. Each predicted contig was grouped into chromosome- or and plasmid-derived (x-axis), coloured based on prediction evaluation and associated probability plasmid-class (y-axis) represented.

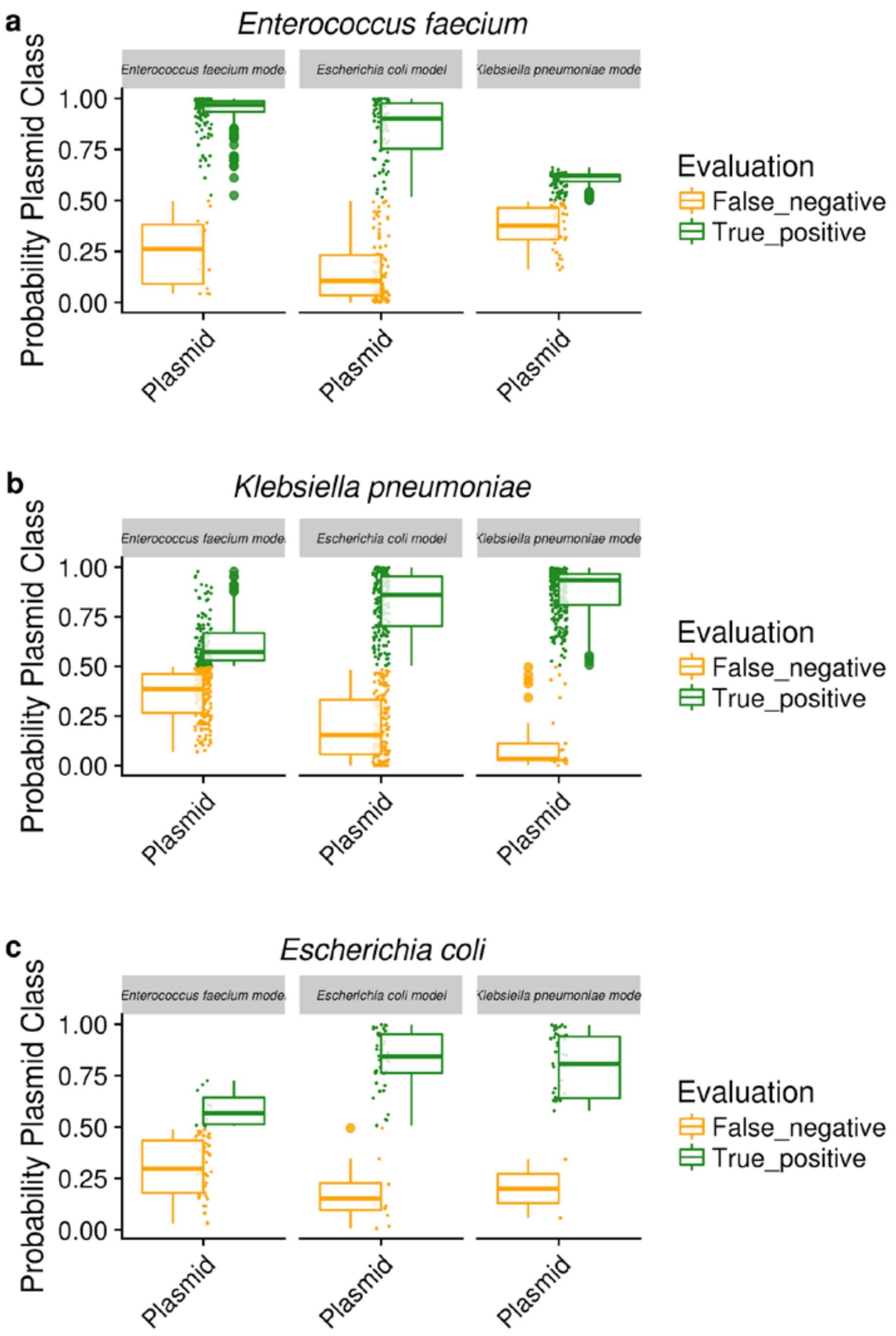

Figure S8. Estimating mlplasmids potential to predict plasmid sequences transferred by HGT events. We used all the three species models available in mlplasmids to predict contigs belonging to *E. faecium* (a), *K. pneumoniae* (b) and *E. coli* (c) validation sets. Each plasmid-derived contig was coloured as false-negative (orange) or true-positive (green) based on evaluation of mlplasmids prediction.

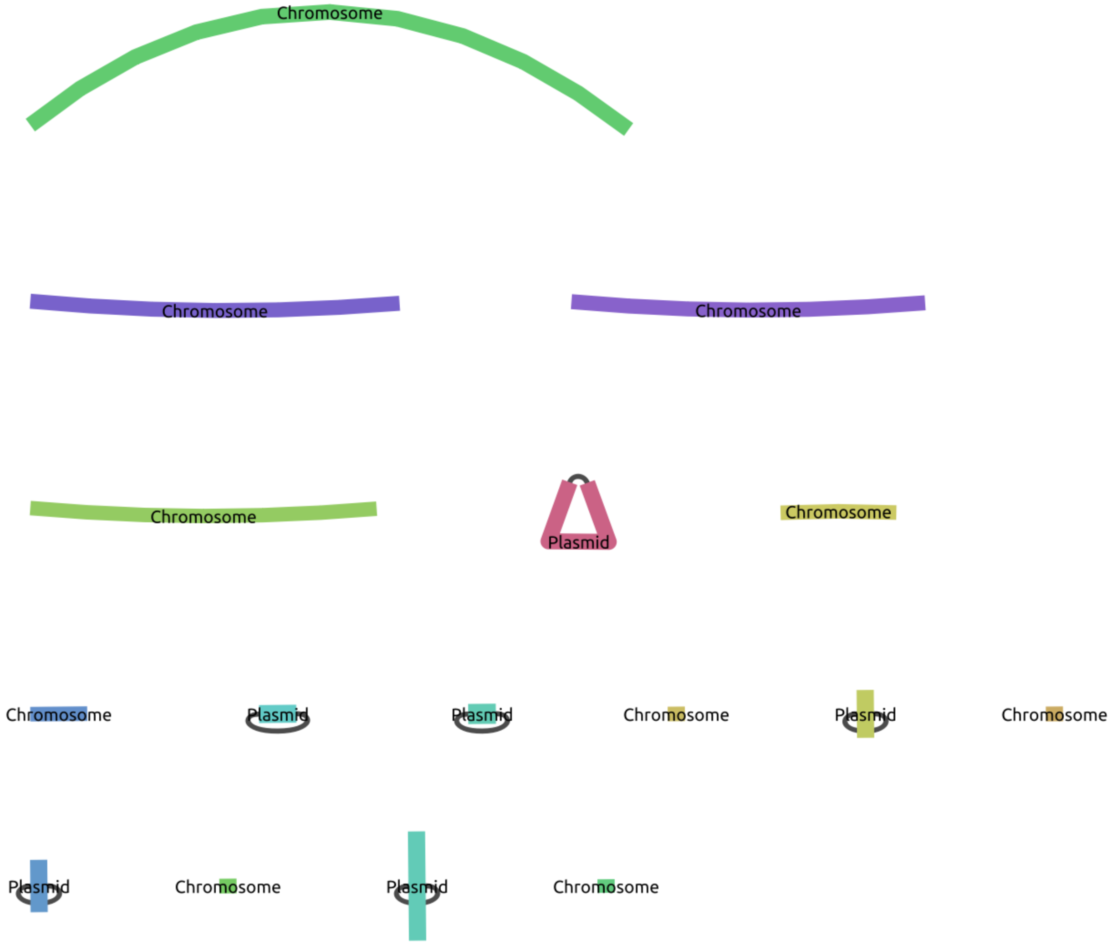

Figure S9. mlplasmids applicability to predict contigs derived from incomplete hybrid or long-read assemblies. Bandage visualization of the hybrid assembly obtained for the *E. faecium* isolate E7070. For this isolate, hybrid assembly using Unicycler did not result in a complete assembly (chromosome and plasmids in single and circular components). Resulting contigs were labeled based on mlplasmids prediction.

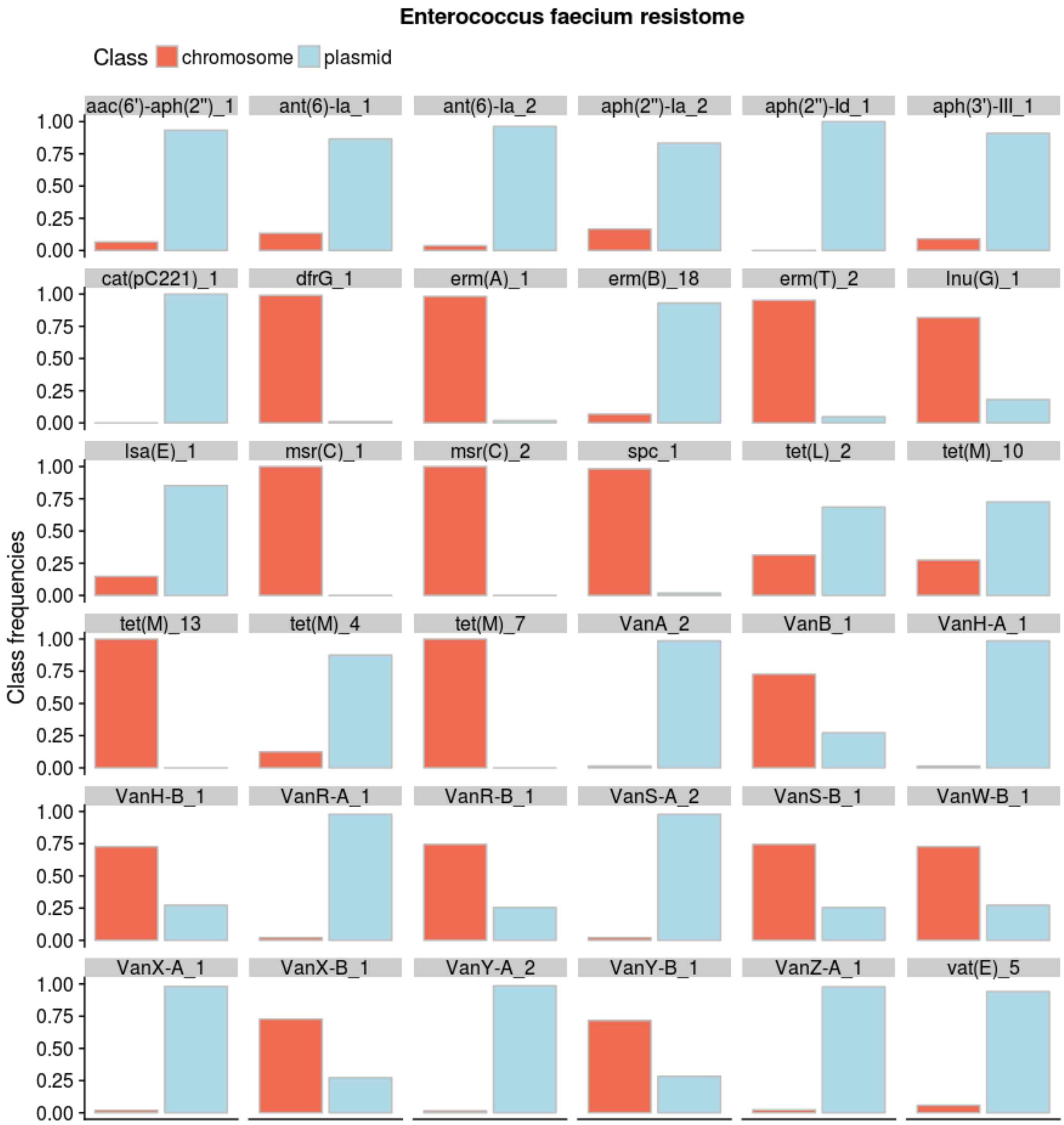

Figure S10. *Enterococcus faecium* resistome. Draft genomes available in NCBI Genomes FTP (n = 369) were downloaded and screened using Abricate and ResFinder for the presence of antibiotic resistance genes. Each contig containing a resistance gene was predicted with mlplasmids to predict plasmid- or chromosome-origin. For visualization purposes, only antibiotic resistance genes present more than five times are shown.

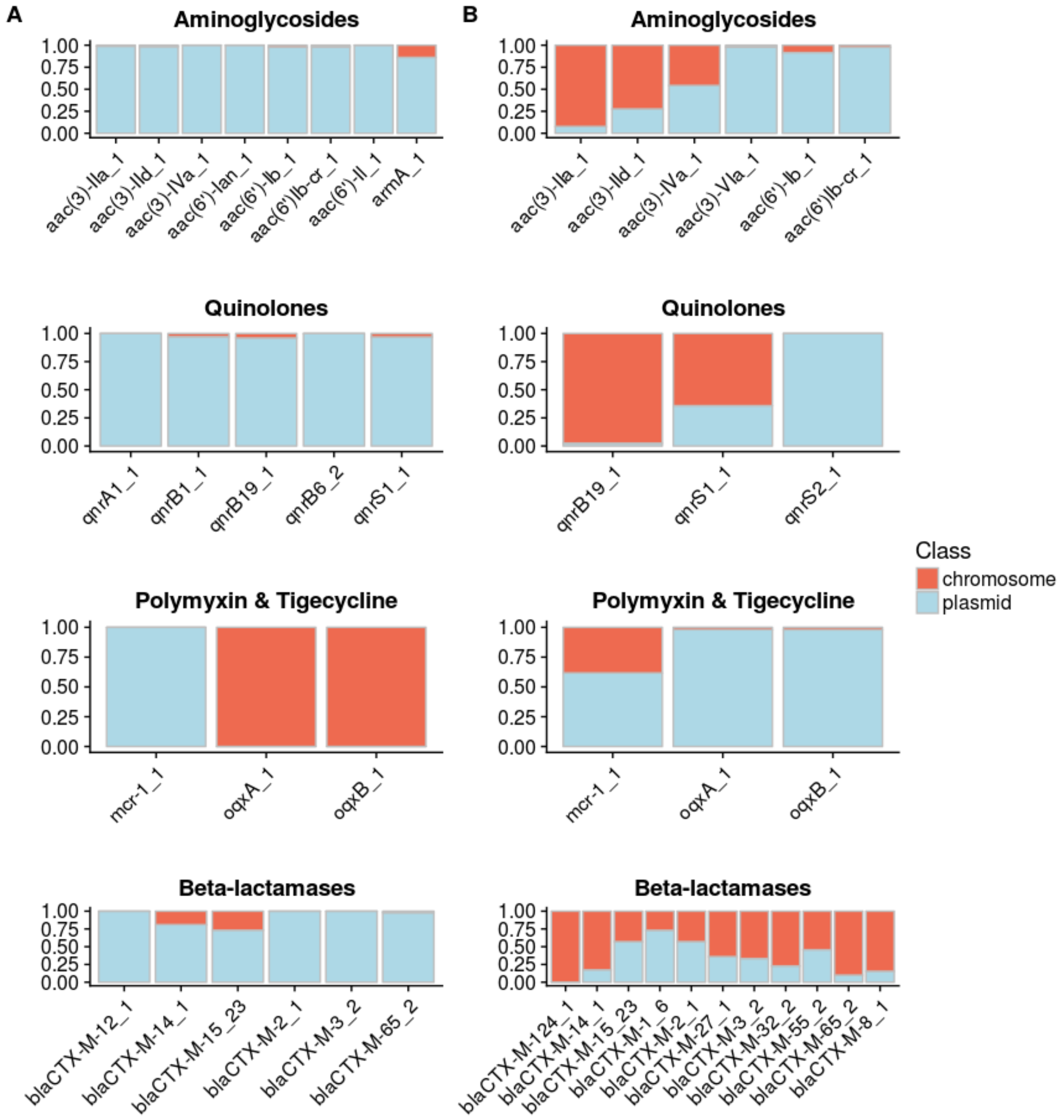

Figure S11. Highlighted genes for *Klebsiella* pneumoniae (panel A) and *Escherichia coli* (panel B).

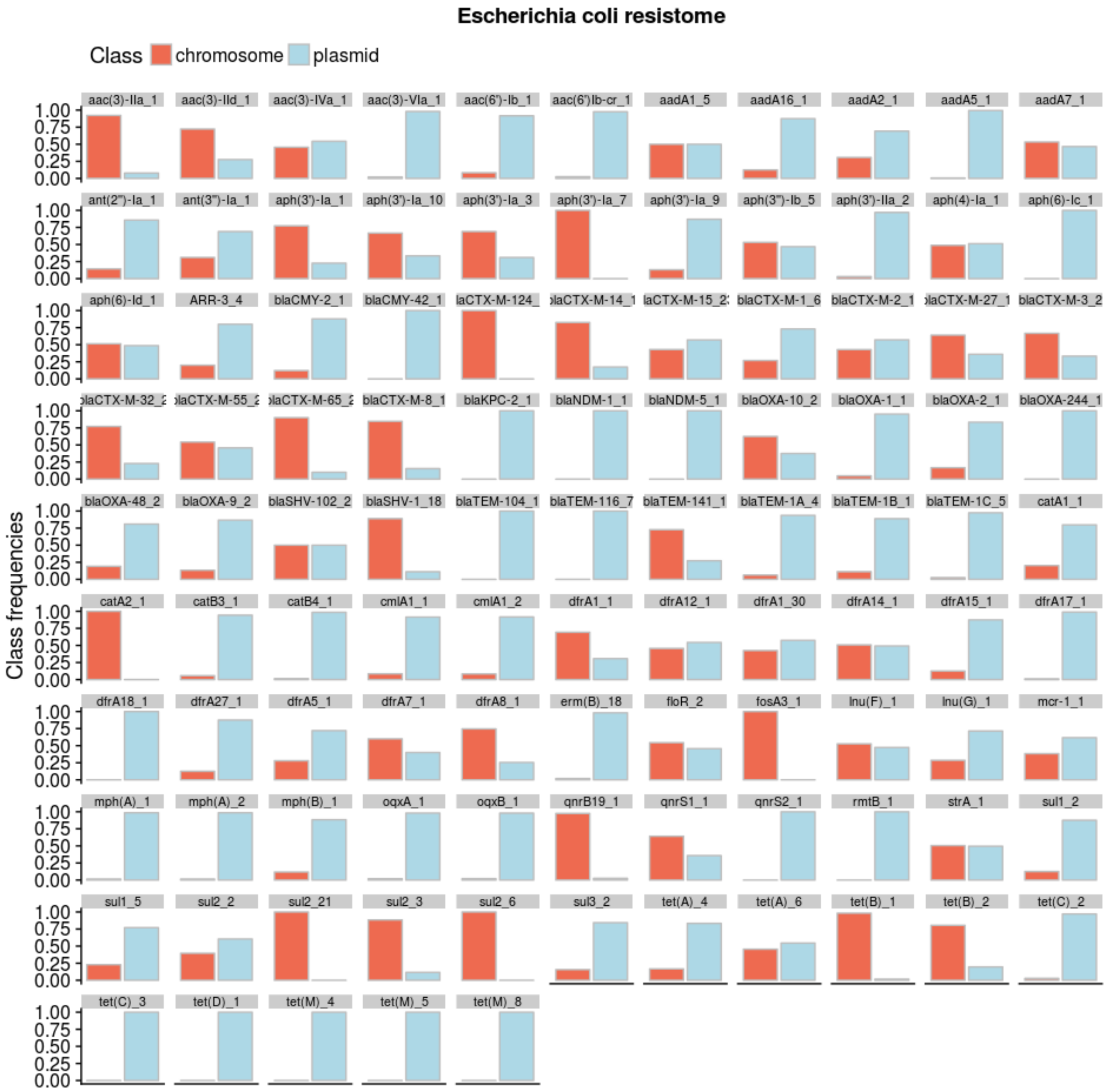

Figure S12. ***Escherichia coli*** **resistome.** Draft genomes available in NCBI Genomes FTP (n = 5,234) were downloaded and screened using Abricate and ResFinder for the presence of antibiotic resistance genes. Each contig containing a resistance gene was predicted with mlplasmids to predict plasmid- or chromosome-origin. For visualization purposes, only antibiotic resistance genes present more than five times are shown.

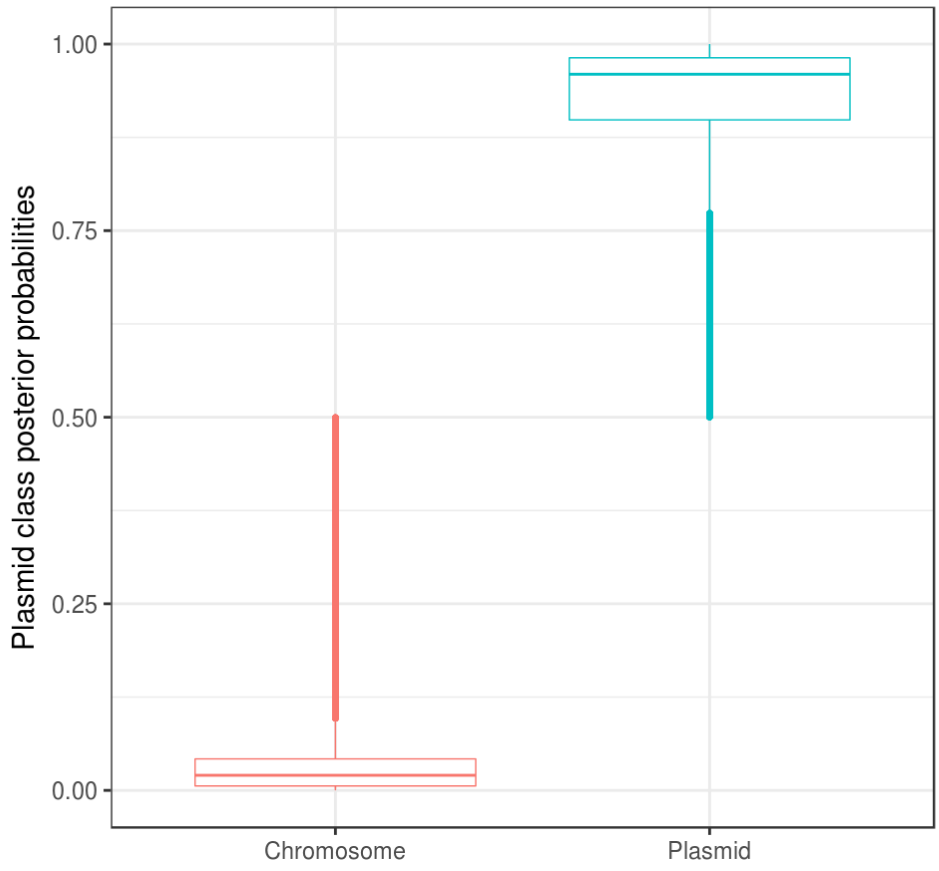

Figure S13. Predicting the plasmidome content of *E. faecium* isolates (n = 1,644). Posterior probabilities of short-read contigs (n= 289,369) of belonging to chromosome- or plasmid-class using our optimized mlplasmids *E. faecium* model for our collection of 1,644 Illumina sequenced *E. faecium* isolates.

References

1. **Antipov D, Hartwick N, Shen M, Raiko M, Pevzner PA**. plasmidSPAdes : Assembling Plasmids from Whole Genome Sequencing Data. *Bioinformatics* 2016;32:3380–3387.

2. **Seemann T**. Prokka: Rapid prokaryotic genome annotation. *Bioinformatics* 2014;30:2068–2069.

3. **Page AJ, Cummins CA, Hunt M, Wong VK, Reuter S, *et al.*** Roary: rapid large-scale prokaryote pan genome analysis. *Bioinformatics* 2015;31:3691–3693.

4. **Maaten L van der, Hinton G**. Visualizing Data using t-SNE. *J Mach Learn Res* 2008;9:2579–2605.

5. **Krijthe J**. Rtsne: T-Distributed Stochastic Neighbor Embedding using Barnes-Hut Implementation (R package version 0.10). *Computer Software*.

6. **Clewell DB, Weaver KE, Dunny GM, Coque TM, Francia MV, *et al.*** *Extrachromosomal and Mobile Elements in Enterococci: Transmission, Maintenance, and Epidemiology*. 2014.

7. **Wick RR, Judd LM, Gorrie CL, Holt KE**. Unicycler: Resolving bacterial genome assemblies from short and long sequencing reads. *PLoS Comput Biol* 2017;13:e1005595.

8. **Bankevich A, Nurk S, Antipov D, Gurevich A a., Dvorkin M, *et al.*** SPAdes: A New Genome Assembly Algorithm and Its Applications to Single-Cell Sequencing. *J Comput Biol* 2012;19:455–477.

9. **Walker BJ, Abeel T, Shea T, Priest M, Abouelliel A, *et al.*** Pilon: an integrated tool for comprehensive microbial variant detection and genome assembly improvement. *PLoS One* 2014;9:e112963.

10. **Bischl B, Lang M, Kotthoff L, Schiffner J**. mlr: Machine learning in R. *of Machine Learning …*. http://www.jmlr.org/papers/volume17/15-066/15-066.pdf (2016).
